# Vimentin Phosphorylation Underlies Myofibroblast Sensitivity to Withaferin A *In Vitro* and during Corneal Fibrosis

**DOI:** 10.1371/journal.pone.0133399

**Published:** 2015-07-17

**Authors:** Paola Bargagna-Mohan, Ling Lei, Alexis Thompson, Camille Shaw, Kousuke Kasahara, Masaki Inagaki, Royce Mohan

**Affiliations:** 1 From the Department of Neuroscience, University of Connecticut Health Center, Farmington, Connecticut, United States of America; 2 Division of Biochemistry, Aichi Cancer Center Research Institute, Nagoya, Japan; Cedars-Sinai Medical Center; UCLA School of Medicine, UNITED STATES

## Abstract

Vimentin is a newly recognized target for corneal fibrosis. Using primary rabbit corneal fibroblasts and myofibroblasts we show that myofibroblasts, unlike fibroblasts, display impaired cell spreading and cell polarization, which is associated with increased levels of soluble serine-38 phosphorylated vimentin (pSer38Vim). This pSer38Vim isoform is inefficiently incorporated into growing vimentin intermediate filaments (IFs) of myofibroblasts during cell spreading, and as a result, myofibroblasts maintain higher soluble pSer38Vim levels compared to fibroblasts. Moreover, the soluble vimentin-targeting small molecule and fibrotic inhibitor withaferin A (WFA) causes a potent blockade of cell spreading selectively in myofibroblasts by targeting soluble pSer38Vim for hyperphosphorylation. WFA treatment does not induce vimentin hyperphosphorylation in fibroblasts. This hyperphosphorylated pSer38Vim species in WFA-treated myofibroblasts becomes complexed with adaptor protein filamin A (FlnA), and these complexes appear as short squiggles when displaced from focal adhesions. The extracellular-signal regulated kinase (ERK) is also phosphorylated (pERK) in response to WFA, but surprisingly, pERK does not enter the nucleus but remains bound to pSer38Vim in cytoplasmic complexes. Using a model of corneal alkali injury, we show that fibrotic corneas of wild type mice possess high levels of pERK, whereas injured corneas of vimentin-deficient (Vim KO) mice that heal with reduced fibrosis have highly reduced pERK expression. Finally, WFA treatment causes a decrease in pERK and pSer38Vim expression in healing corneas of wild type mice. Taken together, these findings identify a hereto-unappreciated role for pSer38Vim as an important determinant of myofibroblast sensitivity to WFA.

## Introduction

Fibrosis is a common outcome to many different types ocular injuries, among which, alkali injuries are some of the most challenging to rehabilitate [1]. In the repairing stroma of injured corneas, resident keratocytes become activated into wound fibroblasts and undergo a differentiation program that converts them into myofibroblasts by acquiring α-smooth muscle actin (α-SMA) expression to form stress fibers for contractile function [2]. This occurs via both paracrine and a feedback autocrine loop involving transforming growth factor (TGF)-β to activate *de novo* expression of α-SMA expression that sustains the myofibroblast phenotype [3] [4]. Fibroblasts develop focal adhesions (FAs) to modulate transmission of forces for their motility that involve both the actomyosin cytoskeleton and the dynamic properties of type III IF, including vimentin [5]. FAs actively engage in cellular processes such as cell spreading and cell migration, wherein vimentin has been shown to govern FA organization in fibroblasts [6] [7]. Myofibroblasts require additional steps to develop mature fibrillary FAs, which is governed by integration of both intracellular and extracellular forces [8] [9].

Vimentin is an evolutionarily conserved cytoskeletal protein that mechanically integrates external stimuli with cellular biochemical processes that control cell structure, shape and movement, by acting together with actin and tubulin to regulate functions of a plethora of cellular proteins [10] [11] [12]. Because its expression is obligatory in tissue remodeling processes such as wound healing, vimentin deficiency leads to inadequate wound repair due to impairment of myofibrobast function [13] [14]. Elsewhere in disease paradigms, vimentin overexpression is observed in numerous types of tumors, and as such, this IF protein has come to be widely studied for its association with pathological disorders [15] [16] [17] [18]. Under normal conditions the majority of cellular vimentin is found as a polymer. Soluble vimentin (sVim), on the other hand, encompasses many vimentin species that include tetrameric subunits to small-sized nonmembrane-bound precursors, where these precursors can become large enough to appear as dots and squiggles by immunofluorescence staining [12]. sVim is generally found at levels below 5–10 percent of the total amount of cellular vimentin in resting cells [19]. Besides being an essential precursor of polymeric vimentin IFs, sVim also has other critical cellular functions. For instance, sVim controls cellular growth signaling pathways acting as a chaperon for mitogen-activated protein kinases (MAPK) (ERK1 and ERK2). Interestingly, ERK1/2 become phosphorylated (pERK1/2) in sciatic nerves upon injury, where it was found that phosphorylated sVim binds and transports pERK1/2 in injured peripheral nerves to promote wound healing [20]. Vimentin-deficient (Vim KO) mice do not display pERK1/2 in injured nervous tissues, illuminating that one critical function of sVim in traumatic injury is to mediate the transport of activated ERK to sites of injury repair [20]. Furthermore, phosphorylated sVim through binding to pERK also protects pERK from dephosphorylation, attesting to an important regulatory function for sVim in growth signaling [21]. In mast cells, sVim also complexes with pERK and p38 MAPK, which extends the idea of that this sVim acts as a critical chaperon for multiple signaling kinases [22]. Vimentin IF polymers exchange soluble subunits dynamically during changes in cellular states [23] [24] when reorganization of nonmembrane-bound precursors, or the entire vimentin IF polymers, is mediated by phosphorylation/dephosphorylation events [25] [26]. This has especially drawn interest to the serine/threonine kinases that govern filament reorganization during cell division and migration [27]. For instance, several mitotic kinases are known to phosphorylate vimentin at its N-terminal region and shown to mediate the depolymerization of vimentin IFs [28] [29]. Vimentin also controls formation of lamellipodia where it is serine 38 phosphoryation that was found to govern localized dismantling of vimentin IFs at the plasma membrane to permit formation of lamellipodia, a process associated with cell polarization and cell motility [30]. Another key N-terminal phosphorylated site on vimentin is serine 56, which mediates smooth muscle cell response to tryptamine-induced p21-activated kinase signaling [31]. As numerous phosphorylation sites have been found on vimentin that cause vimentin IF reorganization and a plethora of kinases as well that can phosphorylate vimentin at multiple sites [32], vimentin phosphorylation is clearly an important mechanism, but how this posttranslational process governs corneal myofibroblast behavior has not been investigated.

Withaferin A (WFA), a steroidal lactone found in the medicinal plant *Withania somnifera*, was discovered as a potent angiogenesis inhibitor [33]. We also found that WFA promotes repair after corneal alkali injury through blocking critical fibrotic mechanisms that are linked to WFA targeting sVim and interfering with vimentin functions [34]. WFA inhibits vascular endothelial cell proliferation in G_0_/G_1_ cell cycle phase (IC_50_ = 12 nM), whereas in comparison, a range of tumor cell lines exhibit growth arrest at considerably higher (average IC_50_ = 250 nM) concentrations [33]. In corneal fibroblasts, WFA induces cell cycle arrest (IC_50_ = 25 nM), mediated by induction of cyclin dependent kinase inhibitor p27^kip1^ expression via downregulation of the ubiquitin E3 ligase Skp2 [34]. This mechanism of growth arrest was also shown to be relevant for inhibition of ocular fibrosis in a glaucoma surgical model of fibrosis [35]. However, when applied in three-dimensional collagen gels, the concentration of WFA required to arrest endothelial cell sprouting [33] [36], which is a measure of invasive cell behavior, or collagen contraction in TGF-β1-treated fibroblasts is significantly higher (IC_50_ = 500 nM), suggesting that major structural functions of vimentin IFs are perturbed at these concentrations. For instance, sub-micromolar concentrations of WFA induce the reorganization of vimentin IFs, causing vimentin IF solubilization to occur at the cell periphery, resulting in the rest of cytoplasmic vimentin IFs that begin to retract into perinuclear aggregates [37] [34] [35]. At higher micromolar concentrations, WFA causes extensive vimentin IF cytoskeleton reorganization and much of the vimentin IF cytoskeleton is also rapidly subject to cleavage into smaller sized fragments that show a pattern typical of calpain-mediated proteolysis [37]. Thus, not only are WFA dose-related effects attributed to different roles of vimentin in the mechano-signaling events engaged during cellular growth events, but also different cell types respond to this drug with varying sensitivities [38] [17] [37]. Given its specificity for binding vimentin WFA has also been exploited as a chemical probe of this protein target [39][40]. As vimentin is overexpressed after corneal alkali injury in stromal myofibroblasts, its increased expression closely parallels the development of fibrosis [34]. WFA treatment in this injury model causes downregulation of vimentin and protects against corneal fibrosis, in part, by attenuating myofibroblast differentiation. Injury to Vim KO mouse corneas show an attenuated fibrotic response with downregulated α-SMA expression in stromal fibroblasts attesting to an important role for vimentin in fibrosis [34].

Here have investigated how phosphorylation of vimentin governs the motile behavior of corneal fibroblasts and myofibroblasts with the aim of identifying novel mechanisms of WFA by which this inhibitor blocks cell spreading. We show that corneal fibroblasts differentiate into myofibroblasts by serial cell passaging in serum media, and these myofibroblasts develop a delayed response to forming vimentin IFs during cell spreading because of increased phosphorylation of vimentin on serine-38 and reduction of serine-71 phosphorylation. This serine-38 phosphorylated vimentin (pSer38vim) isoform is differentially distributed between fibroblasts and myofibroblasts during cell spreading. Thus, WFA treatment also has differential effects on these cell types, causing pSer38vim to become hyperphosphorylated selectively in myofibroblasts, with an ensuing blockade of ERK signaling. Extending our cell culture findings to the corneal alkali injury model of fibrosis, we show that high levels of pSer38vim and activated ERK found in myofibroblasts are potently downregulated by WFA *in vivo*.

## Materials and Methods

### General reagents

WFA was purchased from Chromadex (Santa Ana, CA, USA) and stock solutions were prepared in dimethylsulfoxide (DMSO), aliquoted and stored at −20°C. Cell culture supplies were purchased from Life Technologies (Grand Island, NY, USA), cell culture plates from Thermo Fisher Scientific (Pittsburgh, PA, USA). DMSO and epoxomicin (Epox) were purchased from Sigma-Aldrich (Saint Louise, MO, USA). Blebbistatin (Blebb) was purchased from Tocris Bioscience (Bristol, UK). The vimentin cDNA plasmid was purchased from Origene (Rockville, MD, USA), and antibodies for immunohistochemistry and western blot analysis were purchased from several companies and detail information are provided in each method section.

### Isolation and culture of primary rabbit corneal fibroblasts (RbCFs) and cell treatments

We followed a previously described protocol to culture rabbit corneal keratocytes [34]. Briefly, freshly enucleated eyes (n = 50 eyes) from New Zealand White rabbits (4.75 to 5.75 pounds, approximately 8–12 weeks old of mixed sex) were obtained in saline and shipped overnight on wet-ice shortly after sacrifice from a commercial vendor (Pel-Freez Biologicals, Arkansas, USA). Eyes were washed in sterile saline containing antimicotic/antibiotic agents and any eyes that showed signs of damage to corneas were discarded. Intact corneas were excised and digested with collagenase and primary corneal keratocytes were isolated and cultured in complete DMEM/F12 medium (cDMEM) that contained 10% Fetal Bovine Serum (FBS) and a cocktail of antimicotic/antibiotic agents. Corneal keratocytes were passaged (2 to 8 passage numbers) in presence of 10% serum to induce their differentiation from fibroblasts (RbCF2; passage 2) to myofibroblasts (RbCF8; passage 8). Most studies compared RbCF2 with RbCF8 cells because these reflect their respective phenotypes; intermediate and later passage numbers are indicated where appropriate. Prior to experimental procedures, confluent cell cultures were washed with phosphate-buffered saline (PBS), and incubated with DMEM/F12 medium containing 0.1% FBS for 48 h to induce growth arrest. Then, cells were trypsinized and replated in new culture plates at equivalent cell densities in presence or absence of different concentrations of WFA for indicated times in cDMEM medium. Cells were collected and processed for immunohistochemistry and western blot analysis. In some experiments, cells were also treated with proteasome inhibitor Epox for indicated times. For cell suspension experiments, after serum starvation for 48 h, cells were trypsinized and transferred to non-adherent petri dishes and cultured in presence or absence of different concentrations of WFA in cDMEM medium. Cells were then collected and processed for western blot analysis.

### Transient plasmid transfection of RbCF8 cells and blebbistatin treatment

RbCF8 cells were plated at 60% confluency in glass bottom culture plates and cultured in complete medium for 18 h. Cells were then washed with PBS and cDMEM medium was replaced with serum-free, no antibiotic DMEM/F12 medium for 2 h. GFP-tagged vimentin plasmid cDNA was transfected into cells using Lipofectamine reagents, according the manufacturer protocol. 2 h post-transfection, cells were washed with PBS and allowed to recover 18 h in complete cDMEM medium. The day after, cells were treated for 2 h with blebbistatin (Blebb; 50 μM), washed with PBS and then treated with WFA (500 nM) for 18 h. Endogenous and exogenous vimentin expression was assessed by immunohistochemistry analysis.

### Immunohistochemical analysis

For immunohistochemical analysis of cell cultures, we followed a protocol previously described [34]. Briefly, cells were fixed in ice-cold methanol for 5 min and then in acetone for 1 min at −20°C, blocked and permeabilized in a 0.5% Triton-X/PBS, 5% normal goat serum, 1% BSA solution at 37°C for 1 h and then incubated overnight at 4°C with the following primary antibodies: mouse anti-alpha-smooth muscle actin antibody (α-SMA; clone 1A4; 1:100 Dako North America Inc., Carpinteria, CA, USA), rabbit anti-vimentin antibody (1:100, Abcam, Cambridge, MA, USA), rabbit anti-phospho-vimentin antibody (pSer38Vim, 1:100, Santa Cruz Biotechnology, CA, USA), mouse anti-phospho-p44/42 MAPK (pERK1/2, 1:100, Santa Cruz Biotechnology), and mouse anti-Filamin A (FlnA) (clonePM6/317, 1:100, Millipore, Temecula, CA, USA) in a background reducing solution (Dako North America Inc.). After washing, cells were incubated with goat anti-rabbit IgG secondary antibody Alexa Fluor 488 (1:1000, Life Technologies, Grand Island, NY, USA), or goat anti-mouse IgG secondary antibody Alexa Fluor 555 (1:500, Life Technologies) at room temperature for 45 min. When needed, nuclei were stained with 4,6-diamidino-2-phenylindole (DAPI, 1 mg/mL in 0.1 M PBS) for 10 min at room temperature or phalloidin (1:500, Life Technologies, Grand Island, NY, USA) for 18 h at 4°C. For immunohistochemistry analysis of whole-eye tissues from WT and Vim KO mice, we also followed a protocol previously described [34]. Briefly, frozen eye sections were fixed in 4% PFA for 10 min and then slides were blocked/permeabilized in a 0.5% Triton-X/PBS, 5% normal goat serum, 1% BSA solution and then incubated overnight at 4°C with the following primary antibodies: rabbit anti-phospho-vimentin antibody (pSer38vim, 1:100, Santa Cruz Biotechnology, CA, USA), and mouse anti-phospho-p44/42 MAPK (pERK1/2, 1:100, Santa Cruz Biotechnology). After washing, cells were incubated with the corresponding secondary antibodies as described above. Nuclei were stained with DAPI for 10 min at room temperature. All digital images were acquired on an Olympus IX81 fluorescence microscope equipped with MetaMorph software at the indicated magnification. Images were assembled using Adobe Photoshop Software. To calculate the percentage of long stress fibers in RbCF2 and RbCF8 cells we followed a method previously described [41]. Single images of cells stained with phalloidin were collected. Using Adobe Photoshop Software, a circle (containing the entire cell area) was drawn around each cell and the radius length was calculated. We considered as long stress fiber any phalloidin filament whose length exceeded the radius. To analyze α-SMA staining cells we counted 11 random fields for RbCF2 cells (average of 271 cell nuclei) and 11 random fields for RbCF8 cells (average 150 cell nuclei). To analyze FAs, we collected single images of cells stained with paxillin and the peripheral FA mean area was measured from individual FAs. Using NIH ImageJ software, a free-hand drawing was used to delimit the selected FAs [6], where representative individual FAs (50 to 84) per sample were measured.

### Western blot analysis

For tissue culture experiments, following treatments, cells were gently scraped from plates using a solution of PBS containing 1 mM EDTA and spun down for 5 min. Cell pellets were resuspended in ice-cold Buffer A solution (10 mM HEPES, pH = 7.9, 10 mM KCl, 0.1 mM EDTA, 0.1 mM EGTA, 1 mM DTT, 1 mM PMSF, 1 mM benzamidine, 5 mM NaF, 1 mM spermidine) supplemented with a proteinase inhibitor cocktail (Roche) and left on ice for 15 min. 10% NP-40 solution was added in each tube, vortex for 30 sec and then spun down for 30 sec. Supernatants, representing the cytoplasmic soluble fractions, were collected in separate clear tubes. The remaining pellets were resuspended in Buffer C solution (20 mM HEPES, pH = 7.9, 400 mM NaCl, 1 mM EDTA, 1 mM EGTA, 1 mM DTT, 1 mM PMSF, 1 mM benzamidine, 5 mM NaF, 0.1% NP-40) supplemented with a proteinase inhibitor cocktail (Roche) and rocked vigorously at 4°C for 15 min, and then spun down for 5 min. Supernatants, representing the nuclear fractions, were collected in separate clear tubes. Equal amount of proteins (Bio-Rad protein assay) were subjected to fractionation by sodium dodecyl sulfate-polyacrylamide gel electrophoresis (SDS-PAGE) and transferred to nylon membrane. Protein blots were probed with the following antibodies: rabbit anti-p44/42 MAPK antibody (pERK1/2, 1:2000, Cell Signaling Technology, Danvers, MA USA), rabbit anti-phospho p44/42 MAPK antibody (ERK1/2, 1:2000, Cell Signaling Technology, Danvers, MA USA), mouse anti-vimentin antibody (clone V9, 1:400, SantaCruz, CA, USA), rabbit anti-phospho-vimentin antibody (pSer38Vim, 1:400, Santa Cruz Biotechnology, CA, USA), mouse anti-phospho-vimentin antibody (clone 4A4, pSer55Vim, 1:1000), rat anti-phospho-vimentin antibody (clone TM71, pSer71Vim, 1:1000), rat phospho-vimentin antibody (clone TM72, pSer72Vim, 1:1000), mouse phospho-vimentin antibody (clone MO82, pSer82Vim, 1:1000), rabbit anti-ubiquitin antibody (1:200, Dako North America Inc., Carpinteria, CA, USA), mouse anti-Filamin A (clonePM6/317, 1:100, Millipore, Temecula, CA, USA), rabbit anti-gigaxonin antibody (1:500, Sigma) and mouse anti-β-actin antibody (clone AC-15, 1:20,000, Sigma). Blots were scanned, and band intensities were quantified using NIH ImageJ software and normalized to β-actin levels.

### Immunoprecipitation analysis

RbCF2 and RbCF8 cells were plated in 100 mm culture plates at high cell density (2x10^6^ cells/plate) in cDMEM. 48 h prior experimental procedure, cells were washed with PBS, and incubated with DMEM/F12 medium containing 0.1% FBS. Then, cells were trypsinized and replated in new culture plates in presence or absence of different doses of WFA for 1 h in cDMEM medium. Cells were washed with PBS and soluble protein extracts (400 μl total volume) were collected with an ice-cold buffer containing 20 mM Tris-HCl pH = 7.4, 2% Triton-X, 0.2% SDS, 2 mM EDTA, 2 mM Na_3_VO_4_, 1 mM DTT, 1 mM PMSF, 1 mM benzamidine, 5 mM NaF, supplemented with a proteinase inhibitor cocktail (Roche). Equal amount of protein extracts were pre-cleared using 50 μl of 10% (w/v) Protein A Sepharose beads (Sigma, St Louis, MO, USA) for 1 h at 4°C and then centrifuge at 14,000 rpm for 5 min. Supernatants were transferred into new tubes and 40 ml of phospho-p44/42 MAPK-XP rabbit antibody-sepharose bead conjugated (Cell Signaling Technology, Danvers, MA USA) was added to each tube and incubated overnight at 4°C. The day after, samples were washed 5X with extraction buffer, and immunoprecipitated samples were fractionated by SDS-PAGE. Blots were probed with the following antibodies: rabbit anti-phospho-vimentin 38 antibody (p38Servim, 1:400, Santa Cruz Biotechnology, CA, USA), rabbit anti-p44/42 MAPK antibody (pERK1/2, 1:2000, Cell Signaling Technology, Danvers, MA USA), mouse anti-Filamin A (clonePM6/317, 1:100, Millipore, Temecula, CA, USA) as loading control.

### Ethics statement

All animal experiments were conducted under procedures approved by the Institutional Animal Care and Use Committee of the University of Connecticut Health Center. Mice were housed in specific pathogen free cages in designated laboratory animal housing facilities.

### *In vivo* alkali burn injury model

Alkali injury was performed in corneas of 129Svev wild type and Vim KO mice (both sexes between 1.5 to 4 months of age) essentially as described previously [34]. In brief, mice were anesthetized using a mixture of ketamine/xylazine provided by intraperitoneal injection. Upon cessation of sensory response to toe and ear pinch, a drop of proparacaine was applied to both eyes to induce ocular anesthesia and confirmed with absence of corneal reflex. Mice were subsequently injured by application of 0.15 N sodium hydroxide for 1 min, after which, the eyes were flushed with excess PBS solution. The corneal epithelium from limbus from all round including central cornea was gently removed with a Tooke knife. The injured eye was treated with a drop of Atropine, and subsequently with Tobramycin eye drop followed by Erythromycin eye ointment. Mice were recovered from anesthesia on warming pads until fully ambulatory and returned to housing rooms. Mice treated with tobramycin for two subsequent days post injury and monitored for healthy recovery. Injured mice (n = 8) were treated with vehicle (DMSO) or WFA (2 mg/kg solubilized in DMSO) on the day of injury and every subsequent day by intraperitoneal injection for a period of 7 or 14 days. Mice were sacrificed by carbon dioxide asphyxiation followed by cervical dislocation and eyes enucleated and stored at −80°C for western blot or immunohistochemistry analysis. Non-injured mice were similarly treated for a period of 7 d.

### Gel contraction assay

For gel contraction assays using RbCF2 and RbCF8 cells, we followed a protocol previously described [18]. Briefly, 10^4^ cells were embedded in 100 ml of ice-cold collagen type I solution and plated into a 96-well tissue culture plate (n = 8 gels/treatments). Gels were incubated for 1 h in an incubator at 37°C with 5% CO_2_ to allow polymerization and then recombinant human TGF-β1 (2 ng/ml) (R&D Systems, Minneapolis, MN), in the presence or absence of WFA was added for 2 days. Gels were then analyzed using an inverted microscope and pictures were captured at 2X magnification and gel contractile activity was measured using NIH ImageJ software. Data was subjected to Student’s t-tests.

## Results

### Distribution of vimentin controls filament rearrangement during cell spreading

**Fig 1:**
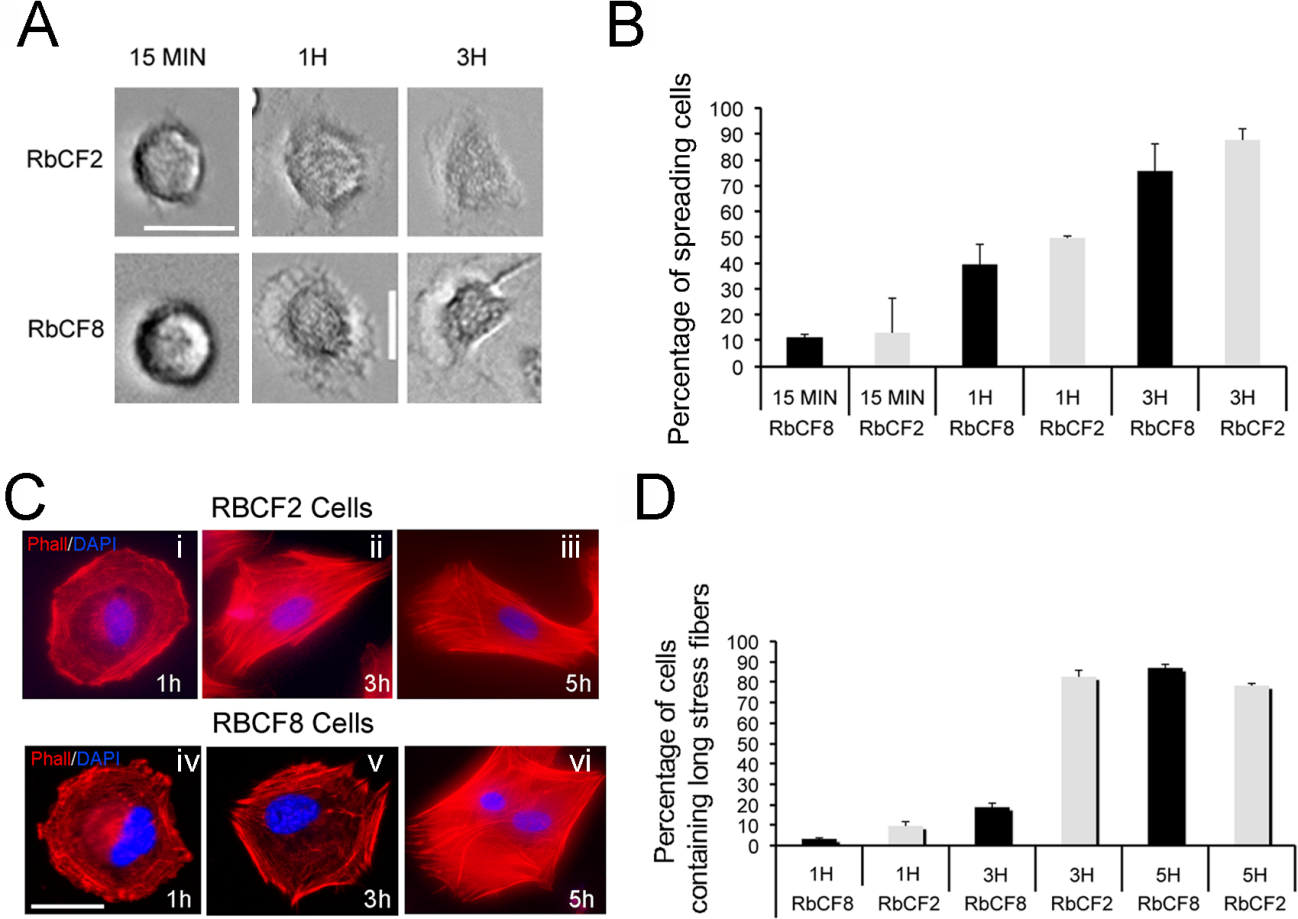
Cell spreading properties depend on cell passage number. RbCF2 and RbCF8 cells were serum starved for 48 h, trypsinized and then replated in presence of 10% serum for different post-plating times. Cells were fixed and cell morphology was analyzed. (A) Representative bright field images of RbCF2 and RbCF8 cells. (B) Graphs represent time-related changes in cell spreading behavior and (D) cell polarization. An average of 70 RbCF8 cells and 154 RbCF2 cells were analyzed. (C) Representative images of RbCF2 and RbCF8 cells at different times after plating and stained with phalloidin (red) and DAPI (blue). Scale bar = 35 μm at 30X magnification.

Cell trypsinization and replating represent an effective *in vitro* model to study the dynamic turnover of IFs, affording changes in the bulk of vimentin IFs [42]. In this study, we used primary rabbit corneal fibroblasts (RbCFs) between passage 2 (RbCF2) and passage 8 (RbCF8), and we focused on early post-plating time points (between 1 h and 3 h) to study cell spreading. We first performed a series of control experiments in which serum-starved cells (from different passage numbers) where trypsinized, replated in 10% serum-containing medium and then analyzed for cell spreading. In fact, the most striking differences were observed between passage 2 (RbCF2) and passage 8 (RbCF8) cells, where their respective phenotypes displayed by the majority of cells (>75%) are represented (Fig 1). The small minor proportions are excluded from the imaging analyses, as they do not account for the relevant phenotypes of these cell populations (investigated further in this study). By phase contrast microscopy analysis we found that both cell types established contact with the substrate within 15 min post-trypsinization (Fig 1A), and cells were able to progressively change shape (Fig 1A). Examination of their stress fiber formation using phalloidin staining showed significant differences in cell spreading behavior (Fig 1C, red). During the first hour post-plating, both RbCF2 and RbCF8 cells displayed radial actin bundles that stained mostly peripherally (Fig 1C, panels i and iv, red). However, over 3 to 5 h post-plating, RbCF2 cells became elongated and formed long stress fibers along the major cell axis, suggesting early cell polarization (Fig 1C, panel ii and iii). RbCF8 cells instead retained largely a radial shape between 1 to 3 h (Fig 1C, panel iv and v, red), with a significant lower amount of long actin fibers compared to RbCF2 cells. Only by 5 h after seeding RbCF8 cells were able to elongate and form actin fibers similar to that of RbCF2 cells (Fig 1C, panel vi). The percentage of cells with which long stress fibers was prominently greater over 1 to 3 h between RbCF2 and RbCF8 cells (Fig 1D).

**Fig 2:**
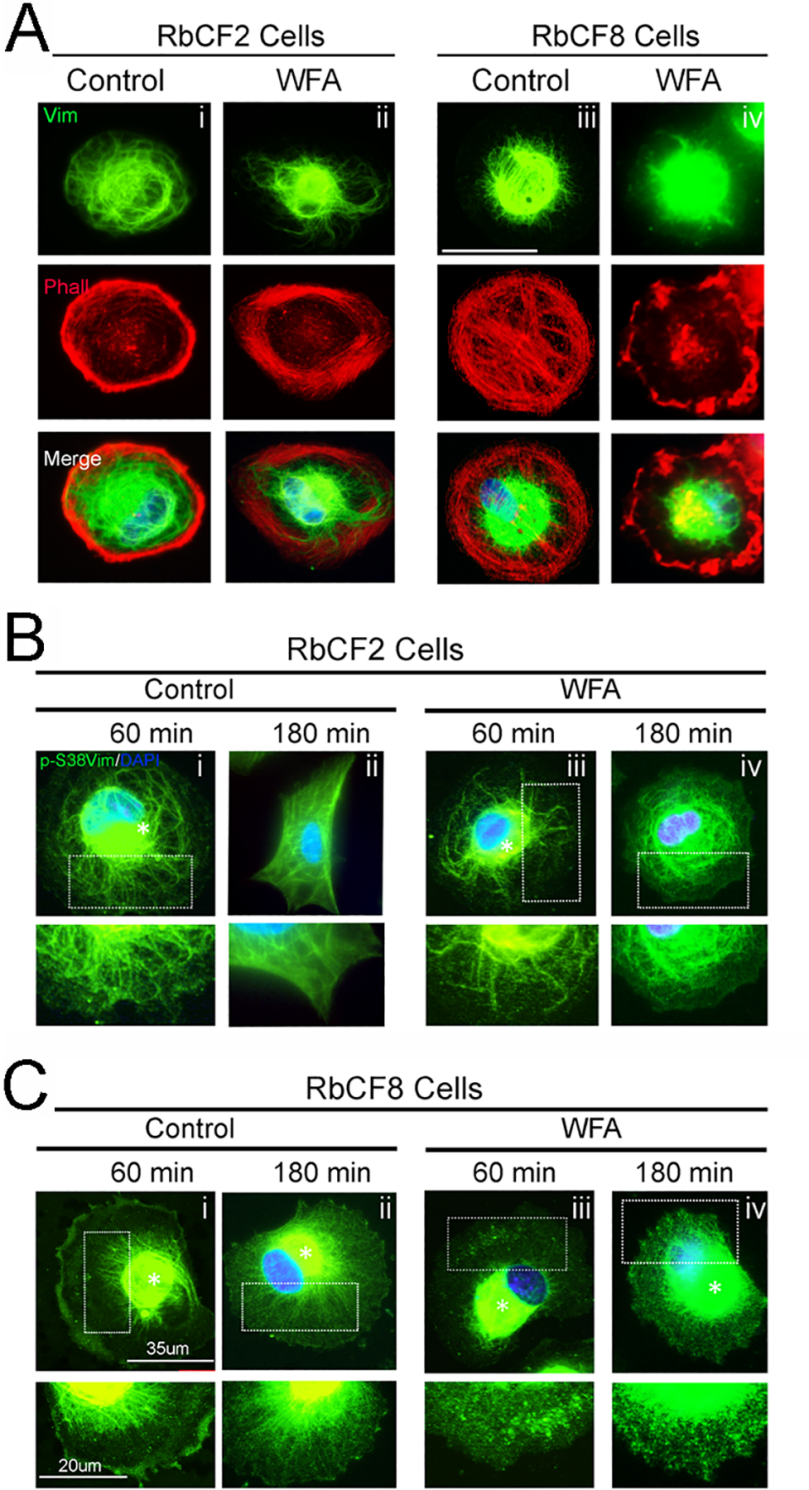
Distribution of vimentin IFs controls filament rearrangement during cell spreading. RbCF2 and RbCF8 cells were cultured as in Fig 1 and plated for 30 min followed by treatment in presence or absence of WFA (1 μM) for different time points. Cells were fixed and stained (A) vimentin (green) and DAPI (blue) or (B-C) with pSer38vim (pSer38vim, green) and DAPI (blue). Scale bar = 35 μm. Insert panels in panels B and C represent 60X magnification of selected areas. Scale bar = 20 μm.

**Fig 3:**
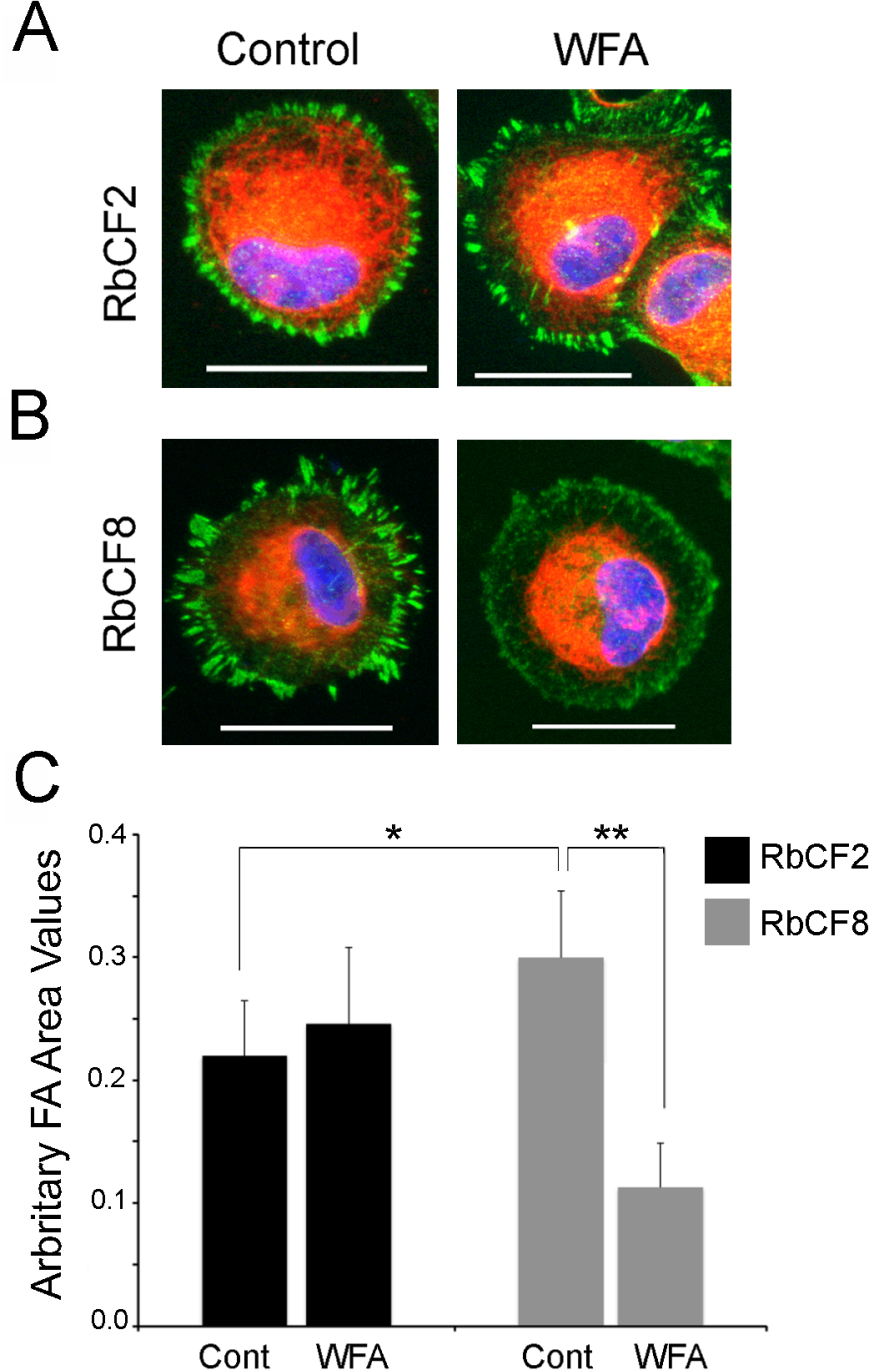
Mature focal adhesions require coalescence with vimentin and develop in a cell-passage dependent manner. (A) RbCF2 and RbCF8 cells were cultured as in Fig 1, plated for 30 min followed by treatment in presence or absence of WFA (1 μM) for 1 h. Cells were fixed and stained with paxillin (green), vimentin (red), and DAPI (blue). Scale bar = 35 μm. (B) Peripheral FA mean areas from RbCF2 (black columns) and RbCF8 (grey columns) cells were measured and represented in arbitrary units. (*P <0.0001; **P <0.0001).

Because RbCF8 cells displayed temporal delay in spreading and vimentin plays a critical role in controlling cell spreading [43], we reasoned that these cells might have an altered vimentin expression profile compared to RbCF2 cells. If this was the case, WFA treatment should differently impact the cell behavior of these cells. We analyzed RbCF2 and RbCF8 cytoskeleton profiles using immunohistochemistry (Fig 2). RbCF2 cells showed a normal distribution of vimentin with filaments radiating from the nuclear envelop to the peripheral cell area (Fig 2A, panel i, green) at 1 h post-plating. WFA treatment only minimally retracted vimentin IFs to the perinuclear zone (Fig 2A, panel ii, green), leaving the actin fiber network intact (Fig 2A, panels i-ii, red). On the other hand, vimentin staining in RbCF8 cells was represented by short, thin IFs, mainly condensed around the nucleus (Fig 2A, panel iii, green). WFA treatment caused increased retraction of vimentin IFs towards the perinuclear area (Fig 2A, panel iv, green) with collapse of the actin network (Fig 2A, panel iv, red). Interestingly, a similar effect was observed when cells were stained for serine-38 phosphorylated vimentin (pSer38vim), which is known to govern vimentin IF stability [30]. In RbCF2 cells, the pSer38vim-stained IFs extended with time to cover the entire cell surface (Fig 2B, panel i), showing a lamella zone enriched with thick pSer38vim IFs and some particles (Fig 2B, enlarged panels from i and ii). WFA did not cause fragmentation of pSer38vim IFs or the formation of dots (Fig 2B, enlarged panels from iii and iv) and there was only minimal pSer38vim perinuclear retraction (Fig 2B, panel iii). RbCF8 cells were unable to branch out their pSer38vim-stained IFs, which remained mainly perinuclear and showed a granular fragmented appearance even at 3 h post-plating (Fig 3C, enlarged panels from i and ii). WFA treatment further retracted pSer38vim network to the perinuclear zone (Fig 2C, asterisk) with abundant accumulation of multiple dots and squiggles in the lamella zone (Fig 2C, enlarged panels from iii and iv). Overall, these results reveal that during early cell spreading events, RbCF8 cells display an impaired pSer38vim-containing vimentin IF network, which would contribute to their heightened sensitivity to WFA during cell spreading (Fig 1).

### Perturbation of vimentin dramatically affects the regulation of mature focal adhesions

Because cell spreading depends on the coalition between vimentin IFs and FAs [6] [43], and RbCF8 cells showed early post-plating impairment in vimentin filamentous structures, we questioned if the delay in cell spreading depended on defects in establishing and growing mature FAs. Synchronized RbCF2 and RbCF8 cells were replated in 10% serum-containing medium in presence or absence of WFA (1 μM) and then stained for paxillin (green) and vimentin (red) (Fig 3). Within 1 h post-plating, RbCF2 cells displayed thick, mature FAs (Fig 3A, left panel, green). WFA treatment caused the appearance of small alterations to FA staining (Fig 3A, right panel, green). We next assessed the peripheral area of FAs [6] in RbCF2 cells and found that WFA caused a small increase (control 0.22 ± 0.05 vs WFA 0.24 ± 0.06; p <0.001). On the other hand, in agreement with results in Fig 2, RbCF8 cells maintained a discoid shape at 1 h post-plating and displayed bundles of filamentous vimentin surrounding the nucleus (Fig 3B, left panel, red). The peripheral area of FAs in RbCF8 cells was also different from control RbCF2 cells (0.32 ± 0.054 vs 0.22 ± 0.05; p<0.0001). Furthermore, WFA treatment of RbCF8 cells further retracted vimentin IFs from the cell periphery, with formation of numerous punctate FAs in the lamella zone (Fig 3B, right panel, green). Consequentially, FAs also became significantly smaller with WFA treatment (0.32 ± 0.054 vs 0.24 ± 0.05; p<0.0001; Fig 3C, grey columns). Taken together, these results reveal that the distribution of vimentin in FAs of RbCF8 cells is altered compared to that in RbCF2 cells, and targeting of developing FAs by WFA depends on vimentin solubility and is cell passage-dependent.

### Forced overexpression of vimentin with myosin perturbation sensitizes cells to WFA

**Fig 4:**
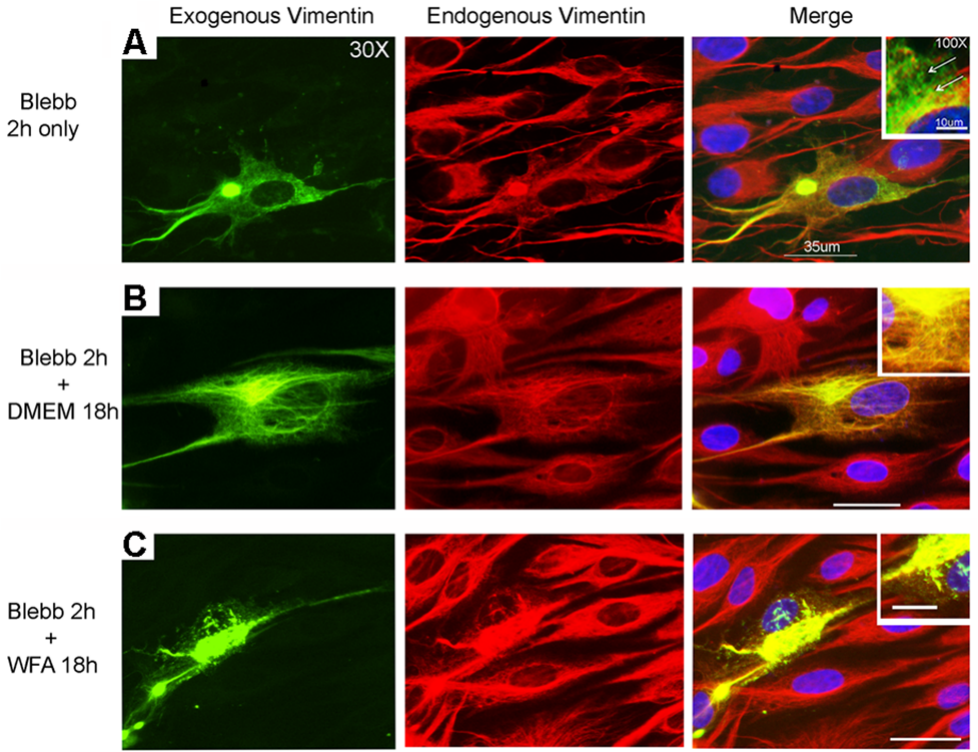
Soluble vimentin is targeted by WFA when overexpressed in RbCF8 cells under myosin perturbation. RbCF8 cells were transiently transfected with human vimentin-GFP cDNA. Two-days post transfection cells were treated with blebbistatin (Blebb, 50 μM) for 2 h (A), washed and then treated in presence (B) or absence of 500 nM WFA (C) for 18 h. Endogenous (red) and exogenous (green; GFP) expression of vimentin was assessed by immunohistochemistry. Scale bar = 35 μm at 30X magnification. Insert panels represent 100X magnification of selected areas. Scale bar = 100 μm.

Next, we wanted to investigate the effects of increasing vimentin expression in fully adherent, well-spread RbCF8 cells and examine how increasing vimentin solubility sensitized cells to WFA. We exploited a recombinant human vimentin-green fluorescent protein (GFP) cDNA to perform transfections in RbCF8 cells (Fig 4). As transfected vimentin readily polymerizes into vimentin IFs from its soluble precursors, we took advantage of blebbistatin (Blebb), an uncompetitive non-muscle myosin II inhibitor, to cause the depolymerization of vimentin IFs [44]. Blebb-treatment alone for 2 h caused significant increase in the sVim pool evidenced by the dot-like appearance of staining (Fig 4A, arrows, enlarged right panel) in cells expressing both exogenous (green, GFP) and endogenous (red) vimentin, whereas in cells expressing only endogenous vimentin, sVim did not increase detectably. Because Blebb’s action is reversible, by performing drug washout after 2 h, we subsequently treated cells with a low dose of WFA (500 nM) and examined vimentin expression after 18 h. Control vehicle-treated cells reacquired their typical filamentous staining pattern for both exogenous human vimentin-GFP and as well endogenous vimentin (Fig 4B, enlarged right panel). However, cells integrating exogenously expressed vimentin with endogenous vimentin were highly sensitive to WFA and displayed extensive prominent dot and squiggle structures (Fig 4C, enlarged right panel). Interestingly, WFA had negligible effects on adjacent neighboring cells that only expressed endogenous vimentin as these did not take up the transfected DNA construct. Taken together, these results identify that sensitivity to WFA in well-spread cells was achieved by increasing sVim levels and through perturbing myosin structures that anchor vimentin to FAs.

### WFA regulates phosphorylation of soluble vimentin in a cell passage-dependent manner

**Fig 5:**
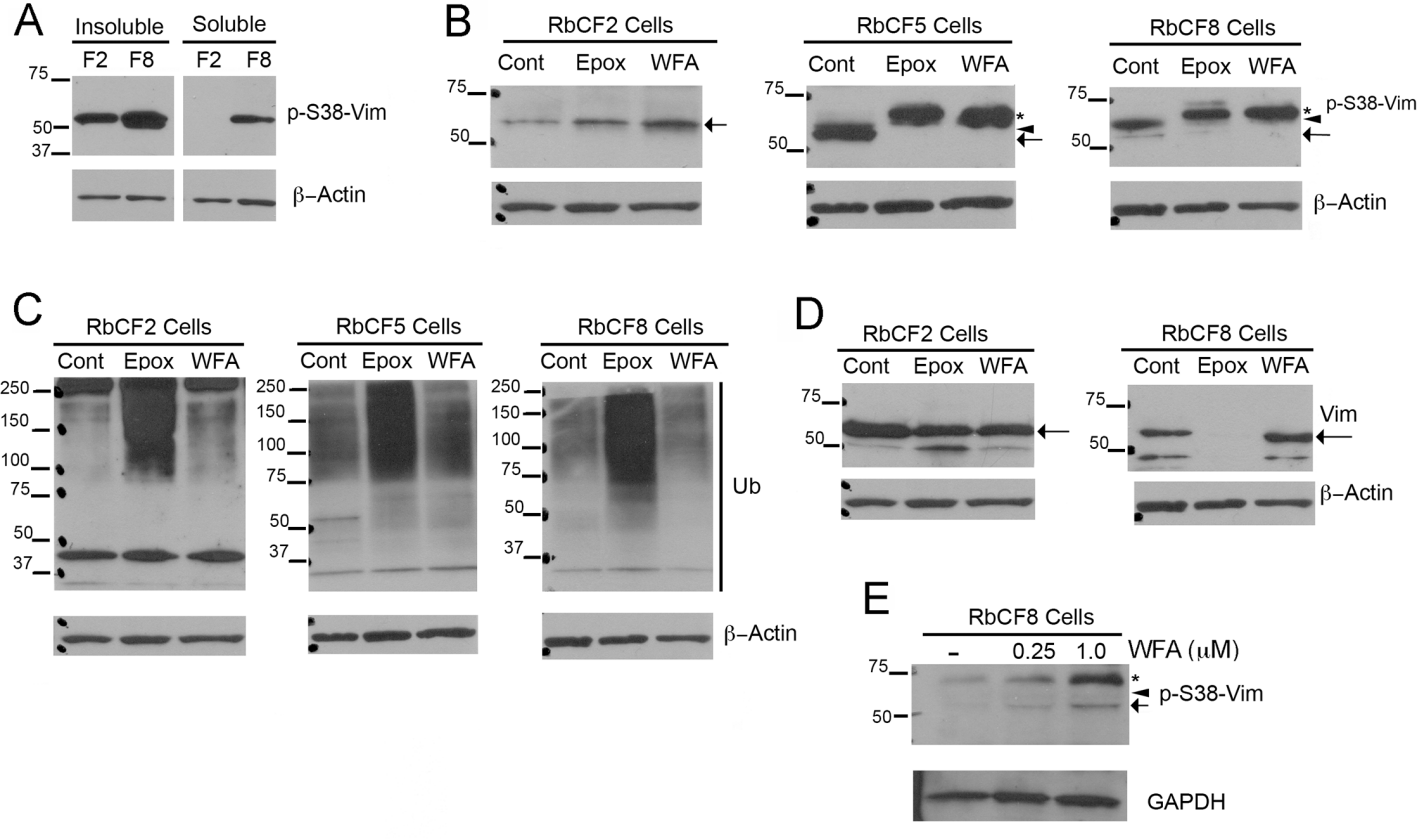
Hyperphosphorylation of soluble pSer38vim by WFA is cell passage dependent. (A) RbCF2 and RbCF8 cells were cultured as in Fig 1 and plated for 1 h. Equal amounts of soluble and insoluble proteins were fractionated by SDS-PAGE and blots were probed with anti-pSer38vim and β-actin antibodies. (B) RbCF2, RbCF5 and RbCF8 cells were cultured as in Fig 1, plated for 30 min and then treated in presence or absence of WFA or Epox for 30 min. Equal amount of soluble proteins were fractionated by SDS-PAGE and blots were probed with anti-pSer38vim and β-actin antibodies. Asterisk marks the 67-kDa high molecular weight pSer38vim hyperphosphorylated species, and the arrow and arrowheads represent the 57 kDa and 61 kDa pSer38vim bands, respectively. (C) Western blots of RbCF2, RbCF5 and RbCF8 cells re-probed with anti-ubiquitin antibody. (D) Western blots comparing sVim expression in RbCF2 and RbCF8 cells cultured as in Fig 1 and treated with Epox or WFA as above. The arrow indicates the major vimentin 57 kDa species. (E) Western blots of RbCF8 cells treated with WFA in suspension culture. Serum-starved cells were trypsinized and transferred into non-adhesive petri dishes in 10% serum-containing medium in presence or absence of WFA for 1 h. Equal amount of soluble proteins were fractionated by SDS-PAGE and blots were probed with anti-pSer38vim and β-actin antibodies. The arrow points to the 57 kDa species, arrowhead to 61 kDa species and asterisk to the 67 kDa species.

**Fig 6:**
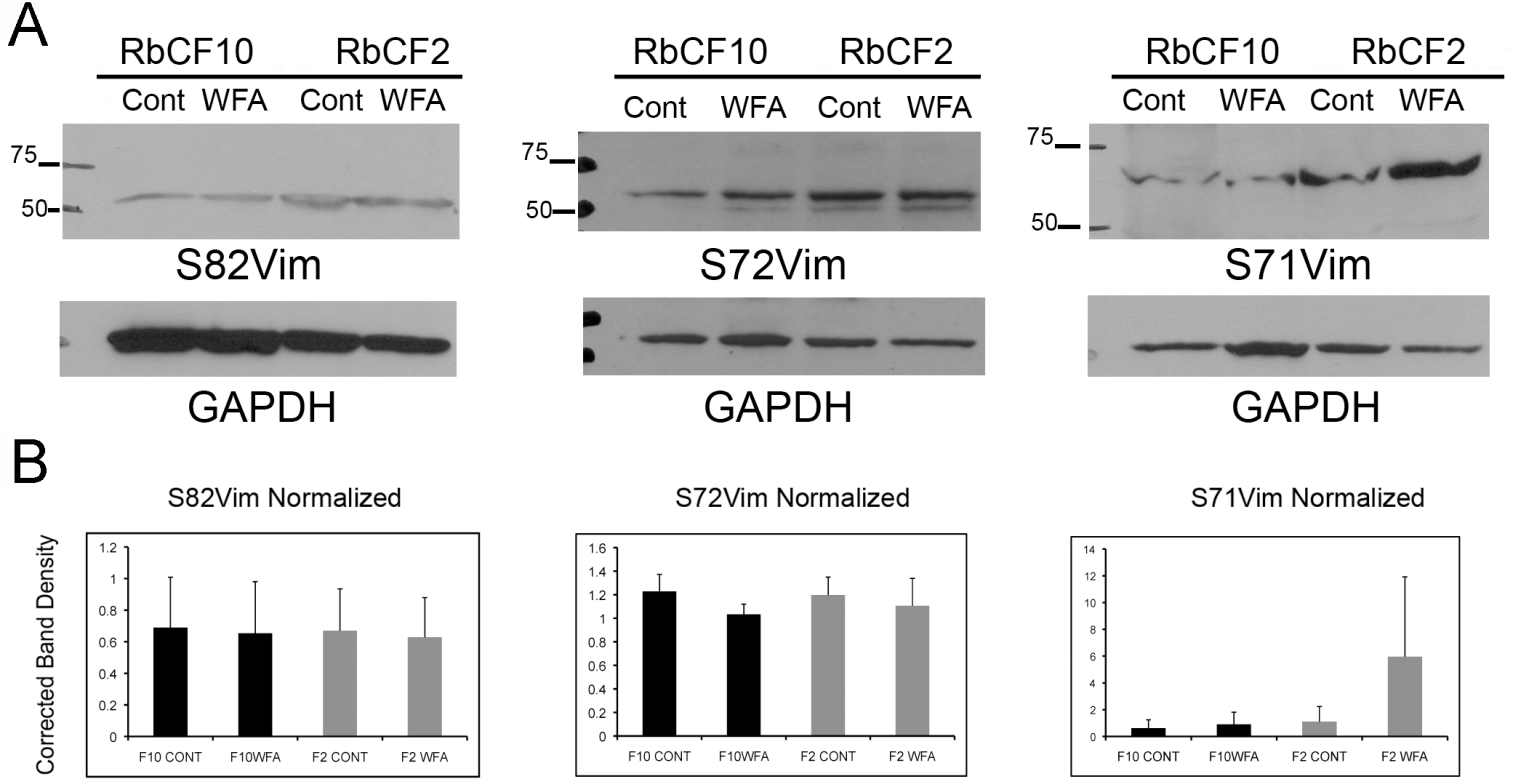
Vimentin phosphorylation at other serine residues. RbCF2 and RbCF8 cells were cultured as in Fig 1, plated for 30 min followed by treatment in presence or absence of WFA (1 μM) for 1 h. (A) Equal amount of soluble proteins were fractionated by SDS-PAGE and blots were probed with anti-S82vimentin (clone MO82), anti-S71vimentin (clone TM71) and anti-S72vimentin (clone TM72), respectively. Blots were then stripped and re-probed for loading controls. (B) Densitometric quantification of repeat experiments (n = 3; see also S1 and S2 Figs) of S82Vim, S72Vim and S71Vim normalized to loading controls, using NIH ImageJ software.

Since it became apparent the differences in the soluble/filamentous form of vimentin in RbCF2 and RbCF8 cells might govern their differential response to cell spreading, we investigated the relative expression of sVim and its phosphorylated (serine 38) forms by western blotting. Serum-starved cells were trypsinized and replated in 10% serum-containing medium to examine RbCF2 and RbCF8 cells for expression of pSer38vim in the soluble and insoluble pools during cell spreading. We found very low levels of pSer38vim in the soluble extracts of RbCF2 cells compared to that from RbCF8 cells at 1 h, whereas the insoluble protein extracts showed abundant levels of pSer38vim in both cell types (Fig 5A). We measured the relative levels of soluble to total amount of pSer38vim (soluble and insoluble) and found that RbCF8 cells retained much higher amounts (20.2%) representing the soluble pool. This vimentin isoform sometimes appeared as a single band (Fig 5A), or as a minor 57 kDa band and major 61 kDa band (Fig 5B; arrow and arrowhead). In comparison, in RbCF2 cells a single 57-kDa band of soluble pSer38vim reaching up to 8.4% of total pSer38vim expression was detected, which corroborated our staining results (Fig 2B). Similar experiments were subsequently performed to assess effects of WFA or the specific proteasome inhibitor epoxomicin (Epox) on protein ubiquitination because corneal keratocytes are known to increase the expression of ubiquitin-proteasome pathway (UPP) proteins during *in vitro* culture when they differentiate into fibroblasts [45]. Serum-starved RbCF2, RbCF5 and RbCF8 cells were trypsinized allowed to attach for 30 min and treated with WFA or Epox for 30 min (Fig 5B) and soluble proteins were analyzed in western blots. In comparison to controls, the higher molecular weight band at 61 kDa was observed in both RbCF5 and RbCF8 cells (Fig 5B; arrowhead). WFA treatment of RbCF2 cells caused levels of the single pSer38vim isoform to increase, whereas in RbCF5 and RbCF8 cells WFA treatment caused both the 57 kDa and 61 kDa pSer38vim bands to run at 67-kDa as a single species (Fig 5B; asterisk). We term this high molecular weight pSer38vim isoform as hyperphosphorylated vimentin. Next, to determine whether additional sites on vimentin show differential phosphorylation, we also examined phosphorylation of serine residues at positions 55, 71, 72 and 82 in early and late passaged cells (Fig 6; S1 and S2 Figs). Serine-71 phosphorylated vimentin (pSer71vim) showed variable levels of induction in response to WFA treatment in RbCF2 cells (average 4.6-fold). Although, late passage cells showed slightly lower basal levels of pSer71vim compared to early passage cells, these differences were not significant nor were pSer71vim levels altered by WFA in RbCF10 cells (Fig 6B; p >0.05). Serine-72 and serine-82 amino acid sites were not phosphorylated in response to WFA from their respective basal levels in both cell types (Fig 6; S1 and S2 Figs). Serine-55 phosphorylation was not detected in western blot analysis (not shown). Importantly, pSer71vim did not show the characteristic WFA-induced increased molecular size to the 67-kDa species as was found with serine-38 modification in late passage cells. Interestingly, this 67-kDa hyperphosphorylated pSer38vim band was similarly produced by Epox treatment in RbCF5 and RbCF8 cells as well (Fig 5B, asterisk), but remarkably, the hyperphosphorylated pSer38vim isoform was not observed in either WFA or Epox-treated RbCF2 cells (Fig 5B, left panel). As anticipated, WFA and Epox differ in their mechanisms of action, which was clearly demonstrated in blots re-probed for ubiquitin (Fig 5C). WFA treatment did not induce expression of polyubiquitinated species in RbCF2, RbCF5 or RbCF8 cells, whereas Epox treatment, as expected, potently increased abundance of polyubiquitinated proteins in all cell types (Fig 5C). Interestingly, Epox treatment also completely abrogated expression of sVim in RbCF8 cells, whereas WFA did not alter sVim levels (Fig 5D). As RbCF2 cells differed dramatically from RbCF8 cells, we focused on these two passage numbers. Finally, to determine whether the hyperphosphorylation of pSer38vim to WFA treatment was influenced by cell adhesion, trypsinized RbCF8 cells were maintained on non-adherent plates for 1 h in presence of serum with and without WFA. Of interest, this culturing condition caused control RbCF8 cells to switch the relative distribution of soluble pSer38vim species showing higher levels of the 67-kDa band compared to minor presence of the 57 kDa species. However, under WFA treatment both bands were retained and their relative abundance increased, with the major change being observed in the 67-kDa species (Fig 5E). Collectively, these results show that soluble vimentin undergoes differential phosphorylation in corneal fibroblasts when cultured *in vitro*, with increasing number of cell passages enhancing their sensitivity to WFA through an increase in levels of pSer38vim isoforms. Furthermore, even though under non-adherent conditions myofibroblasts express basal levels of the 67-kDa band as the major pSer38vim species, this vimentin species remains sensitive to WFA activity. This suggests that cell adhesion is not a requirement for the WFA-induced phosphorylation event. Together, these data suggest that WFA binding to soluble vimentin causes pSer38vim-containing species to become hyperphosphorylated and remain soluble.

### WFA alters Filamin A organization in late passage cells

**Fig 7:**
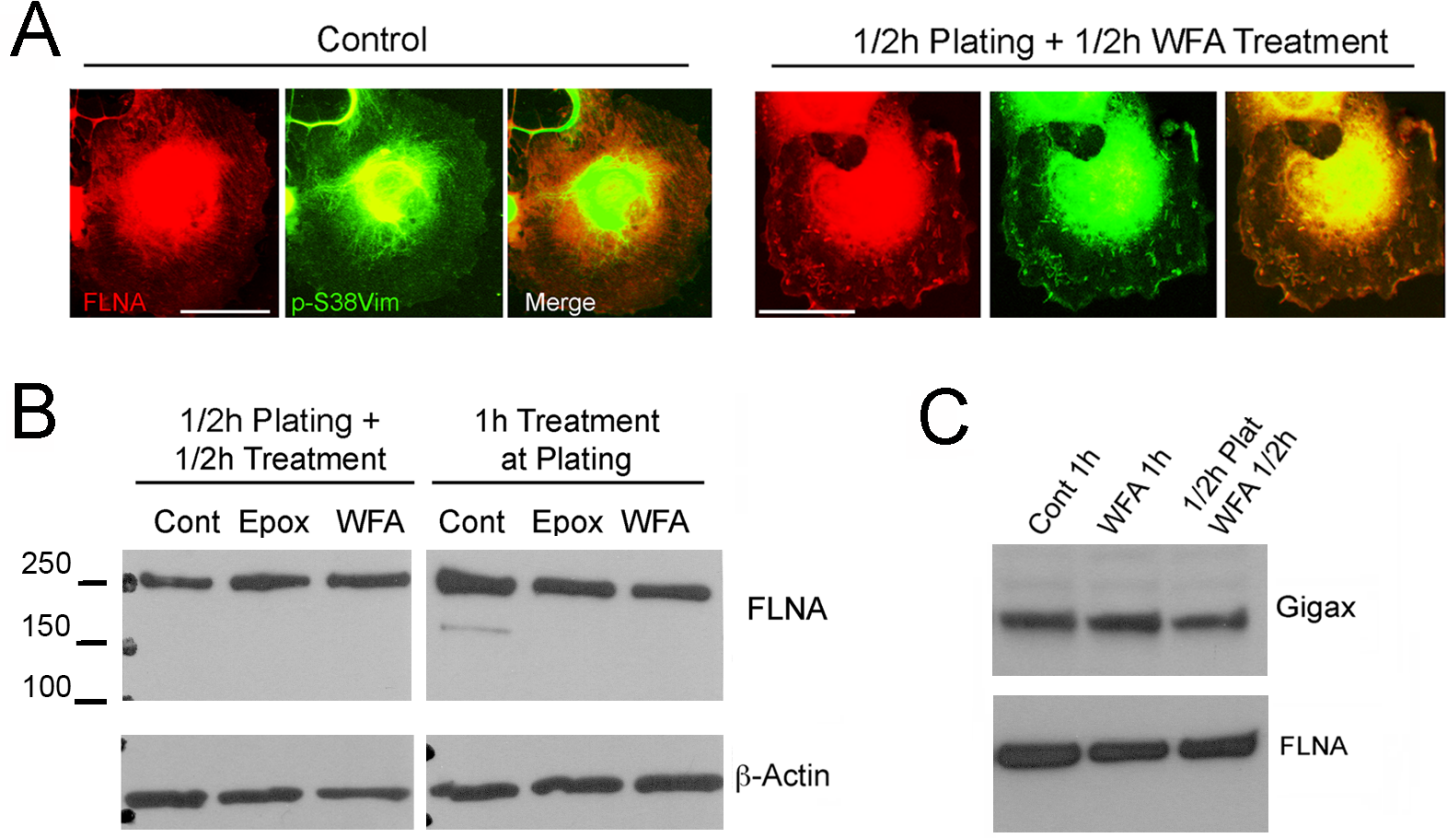
WFA alters Filamin A organization but not its expression. (A) RbCF8 cells were cultured as in Fig 1 in presence or absence of WFA or Epox for 30 min. Control and WFA-treated cells were fixed and stained with anti-pSer38vim (green) and anti-FLNA (red) antibodies. Scale bar = 35 μm at 30X magnification. (B) Control, WFA and Epox-treated cells cultured with drug at time of plating for 1 h (Right Panels) or cells allowed to attach for 30 min and then treated with WFA or Epox for additional 30 min (Left Panels). Equal amount of soluble proteins were fractionated by SDS-PAGE and blots were probed with anti-FLNA antibody. β-actin antibody was used as loading control. (C) Serum-starved cells were trypsinized and replated in 10% serum-containing medium in presence or absence of WFA for 1 h or allowed to attach for 30 min and then treated with WFA for additional 30 min. Equal amount of soluble proteins were fractionated by SDS-PAGE and blots were probed with anti-FLNA and anti-gigax antibodies.

Since our findings showed that pSer38vim complexed with FlnA, we wondered whether WFA affects the subcellular distribution of the vimentin-FlnA complexes in RbCF8 cells (Fig 7). Cells were serum starved, replated and then treated in presence or absence of WFA for 1 h, and then processed for immunohistochemistry. As expected, control RbCF8 cells revealed FlnA expression (Fig 7A, red) along stress fiber orientation, whereas the staining of pSer38vim (Fig 7A, green) was highly punctate and diffused filling the entire cytoplasm till the cell periphery. Overlap between pSer38vim and FlnA was observed only at the perinuclear region where bulk of the cell periphery showed distinct staining (Fig 7A, merge). Cells treated with WFA displayed cytoplasmic pSer38vim staining that had a distinct squiggle-like shape morphology found in the lamella zone (Fig 7A, green). Interestingly, WFA also altered FlnA distribution, which was now found associated with the squiggle structures co-localized with pSer38vim (Fig 7A, red). These short squiggles appeared of similar size and thickness and were distributed randomly throughout the cytoplasm. Western blot analysis of cells treated similarly showed that WFA did not effect changes in FlnA protein expression levels, whether the drug was provided either at time of plating or 30 min after plating when cell adhesion had occurred (Fig 7B). Similar results on FlnA expression were obtained upon treatment with the proteasome inhibitor Epox. Moreover, it was recently shown that gigaxonin (gigax) is a vimentin-specific E3 ligase that targets vimentin [46]. Therefore, we also checked if gigax levels became altered in RbCF8 cells treated with WFA and found that neither WFA treatment at plating or 30 min after plating changed the basal levels of gigax (Fig 7C). These findings reveal that WFA does not affect gigax expression during cell spreading. Taken together, these data indicate that WFA causes specific changes in the cellular distribution of pSer38vim/Fln A-containing protein-protein complexes in RbCF8 cells that could impair the formation of mature FAs.

### Cell contractile properties are differentially regulated in early and late passage cells

**Fig 8:**
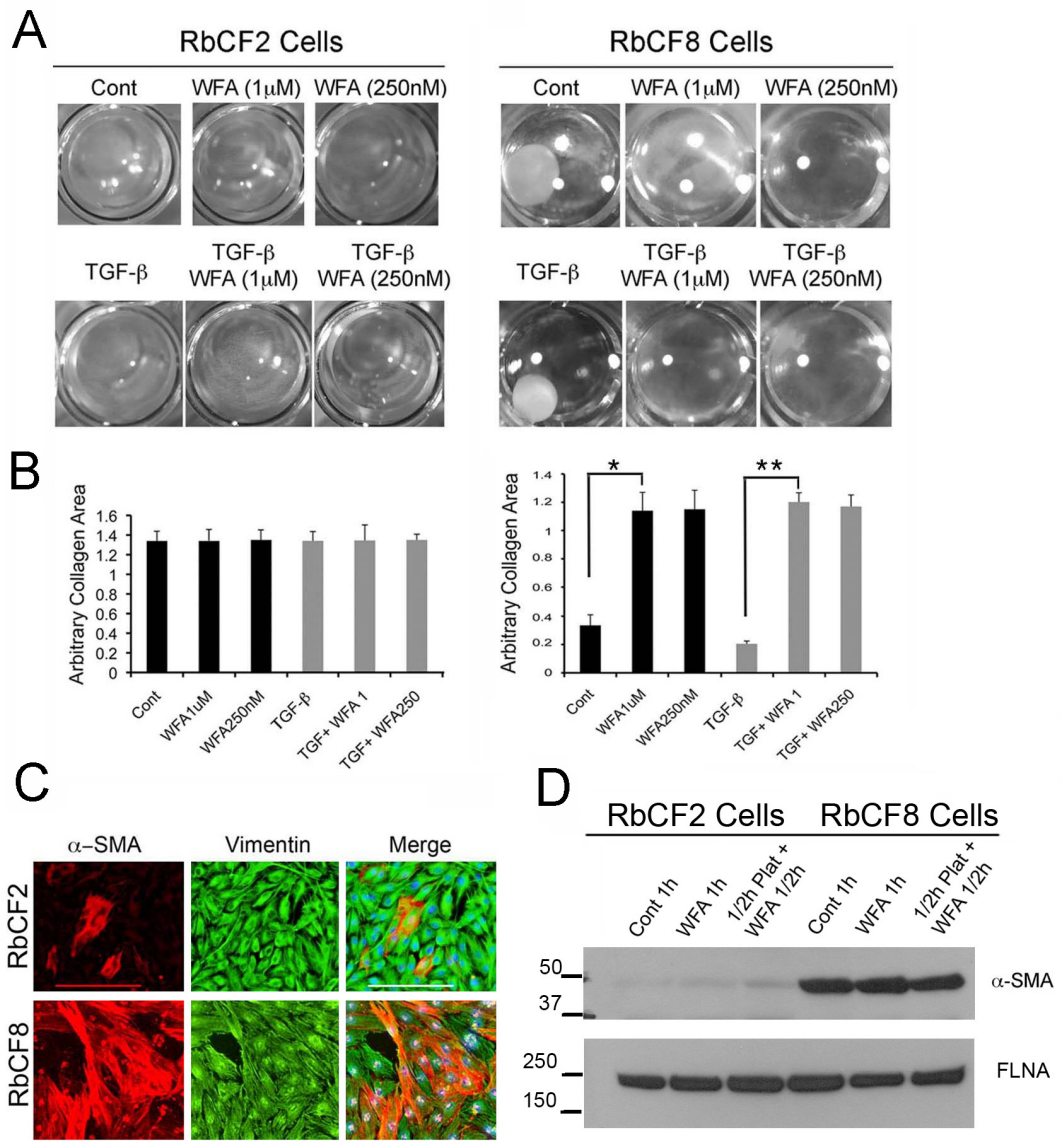
WFA controls gel contraction properties in a cell-passage dependent manner. (A) Representative images of RbCF2 and RbCF8 cells embedded in collagen type I gels and treated with TGF-β1 (2 ng/ml) for 2 days in presence or absence of different doses of WFA. Gels were then fixed and images were captured at 2X magnification. (B) Quantification of gel contractile activity. Images from (A) were used to arbitrarily measure the collagen area in each well using ImageJ program (n = 8 wells/treatment). (C) Immunohistochemistry of RbCF2 and RbCF8 cells cultured on slides and stained with α-SMA (red), anti-vimentin (green) and DAPI (blue). Scale bar = 215 μm at 10X magnification. (D) Western blot analysis of RbCF2 and RbCF8 cells for α-SMA expression. Cells were trypsinized and plated for 30 min and then treated with 1 μM WFA for 30 min or treated with WFA at time of plating for 1 h. Blots were probed with anti-α-SMA and FLNA antibodies used as loading control.

In our previous studies we showed that WFA antagonizes TGF-β signaling in corneal fibroblasts [35]. Following on those results, we investigated if there was a correlation between cell spreading defects and TGF-β-induced cell contraction properties of early and late passage cells. RbCF2 and RbCF8 cells were embedded in a collagen matrix and treated in presence or absence of TGF-β1 with WFA. Notably, TGF-β1-induction of myofibroblast transformation promotes gel contraction within 5–7 days post-treatment. Interestingly, RbCF8 cells within 2 days in culture contracted collagen gels even in absence of TGF-β1, identifying their mature myofibroblast phenotype (Fig 8A). Notably, WFA prevented collagen contraction in absence of TGF-β1 (3.4-fold reduction; p < 0.0001) and in the presence of TGF-β1 (5.8 fold reduction; p < 0.0001) (Fig 8B). Notably, given the same 2-day time frame, RbCF2 cells were unable to display any contractile activity even in presence of TGF-β1. Given the increased contractile properties of RbCF8 cells, we wished to ascertain the percentage of RbCF8 cells that showed α-SMA expression compared to RbCF2 cells (Fig 8C). In fact, as assessed by counting the numbers of α-SMA-positive cells, RbCF8 cells had acquired significantly greater numbers of myofibroblasts than RbCF2 cells (mean 77% ± 11.5 vs 23% ± 4.5; p<0.0001). This data was further corroborated by western blot analysis that showed RbCF8 cells had 8-fold higher levels of α-SMA expression compared to similarly cultured RbCF2 cells (Fig 8D). Importantly, in these adherent cultures WFA treatment did not alter α-SMA expression during cell spreading (whether added at the time of plating or 30 min post-plating), demonstrating that α-SMA is not directly targeted by this vimentin-inhibitor. Taken together, we found that the vast majority of RbCF8 cells exhibit characteristics of mature myofibroblasts with increased α-SMA expression and exert strong cell contractile properties even in absence of exogenous TGF-β1, whereas RbCF2 cells having very low levels of α-SMA, did not exhibit the contractile function of myofibroblasts.

### WFA perturbs the MAPK/ERK signaling pathway during cell spreading in late passage cells

**Fig 9:**
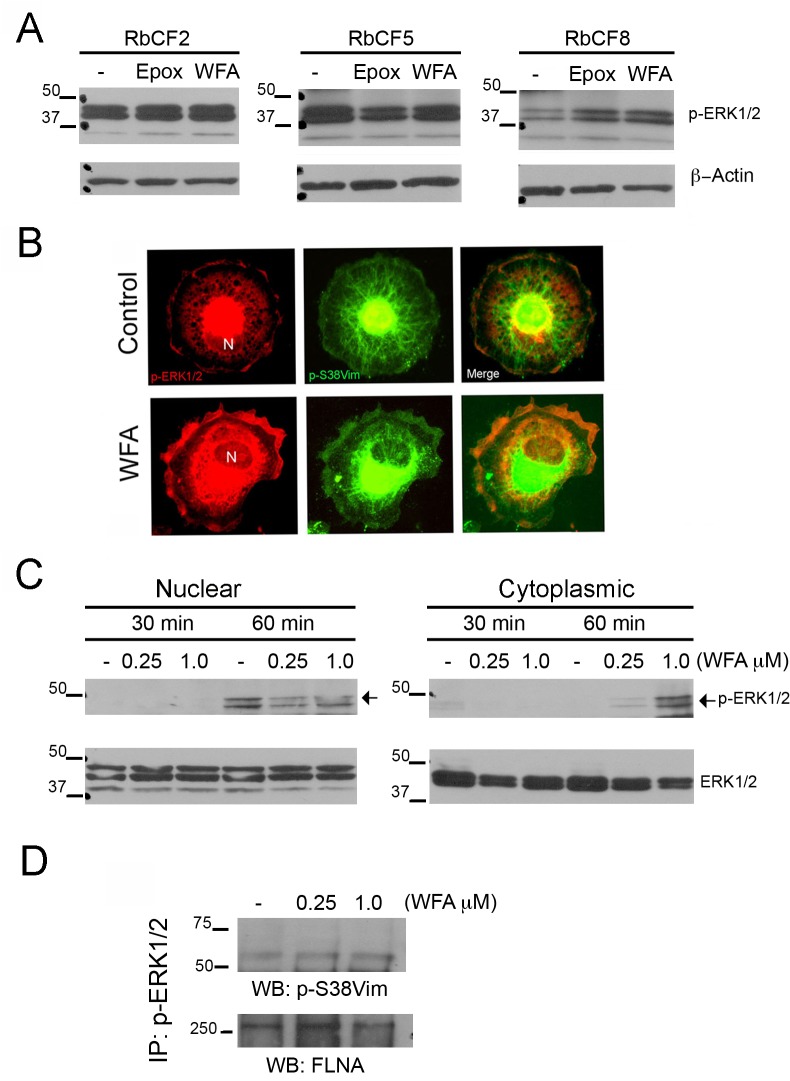
ERK Activation is cell passage dependent. (A) Western blot analysis of RbCF2, RbCF5 and RbCF8 cells. Cells were cultured as in Fig 1, plated for 30 min and treated in presence or absence of WFA and Epox for 30 min. Equal amount of soluble proteins were fractionated by SDS-PAGE and blots were probed with anti-pERK1/2 and β-actin antibodies. (B) RbCF8 cells were cultured as in Fig 1, plated for 30 min and treated in presence or absence of WFA for 30 min. Cells were fixed and stained for pSer38vim (green) and pERK1/2 (red). N = Nucleus. Scale bar = 35 μm at 30X magnification. (C) Western blot analysis of RbCF8 cells. Serum-starved cells were trypsinized, plated for 30 min and then treated with WFA (250 nM and 1 μM) for indicated times. Equal amount of nuclear and cytoplasmic extracts were fractionated by SDS-PAGE and blots were probed with anti-pERK1/2 antibody. Blots were then stripped and reprobed with anti-ERK1/2 antibody as loading control. (D) Immunoprecipitation analysis of RbCF8 cells. Cells were serum-starved 48 h, trypsinized and then replated in presence or absence of different doses of WFA. Equal amount of soluble cell lysates were immunoprecipitated with anti-pERK1/2 antibody and then probed with anti-pSer38vim antibody. Filamin A (FlnA) was used as loading control.

Given that phosphorylated sVim binds to pERK and acts as a chaperon for this kinase, ERK represents an important signaling node that could mediate downstream effects of the MAPK/ERK pathway in cell spreading events [11]. We therefore assessed expression and activation of ERK in RbCF2, RbCF5 and RbCF8 cells by western blot analysis. RbCF2 and RbCF5 cells did not show any perturbation of pERK1/2 expression in the presence of WFA (Fig 9A). However, RbCF8 cells when treated with WFA showed an increase in ERK activation compared to control cells, which was revealed by expression of phosphorylated forms (pERK1/2). Intrigued by these results, we investigated pSer38vim and pERK1/2 staining in RbCF8 cells by immunofluorescence staining. Serum-starved cells were trypsinized, allowed to attach to the substrate for 30 min and treated with WFA for 30 min. Control cells showed pERK1/2 staining mainly concentrated in the nucleus (Fig 9B, red, upper panel) and a fragmented, perinuclear pSer38vim network (Fig 9B, green, upper center panel). Interestingly, in WFA-treated cells pERK1/2 staining was mainly localized in the cytoplasm and at the cell periphery (Fig 9B, red, lower panel), with fragmentation of the pSer38vim network with formation of dots and squiggles and perinuclear condensation (Fig 9B, center lower panel, green). To further examine this mechanism of WFA, RbCF8 cells were treated similarly and analyzed by western blotting for nuclear and cytoplasmic abundance of pERK1/2. Spreading control cells showed a time-dependent nuclear increase in pERK levels with corresponding decrease in cytoplasmic abundance between 30 min and 1 h (Fig 9C, left panel). These changes were not associated with total ERK protein levels, suggesting that only the activated form of ERK1/2 was redistributed in RbCF8 cells. Importantly, WFA in a dose-dependent manner reduced the nuclear accumulation of pERK1/2 and elicited increased pERK1/2 expression in the cytoplasmic extracts (Fig 9C). WFA treatment also did not cause changes in expression levels of total ERK1/2, revealing that WFA affected activated ERK1/2 and promoted its novel subcellular distribution. Finally, we next tested whether binding of pSer38vim by WFA that caused its hyperphosphorylation in RbCF8 cells could interfere with the formation of protein complexes containing pSer38vim-pERK1/2. Cytoplasmic extracts from control and WFA-treated RbCF8 cells were immunoprecipitated with anti-pERK1/2 antibody and protein blots probed with anti-pSer38vim antibodies (Fig 9D). The levels of pSer38vim in immunoprecipitates were low in control RbCF8 cells but showed a dose-dependent increase in response to WFA treatment. As vimentin was shown to bind FlnA, and pERK-FlnA interactions have also been demonstrated in other systems [47], we examined the levels of FlnA in immunoprecipitated pSer38vim-pERK1/2 protein complexes. FlnA was present in the immunoprecipitated complexes from RbCF8 cells, but no significant change in levels of pERK-bound FlnA was detected between the treatment groups. Collectively, these results reveal that the binding of WFA to pSer38vim causes its distribution to be affected but does not disrupt protein-protein interactions in the ligand-bound multimeric pSer38vim-pERK1/2-FlnA protein complexes. Instead, WFA limits the nuclear abundance of pERK1/2 and through this mechanism interferes with pERK1/2.

### WFA treatment and vimentin deficiency attenuate injury-induced activation of the extracellular-signal-regulated kinase 1/2 (ERK1/2) pathway

**Fig 10:**
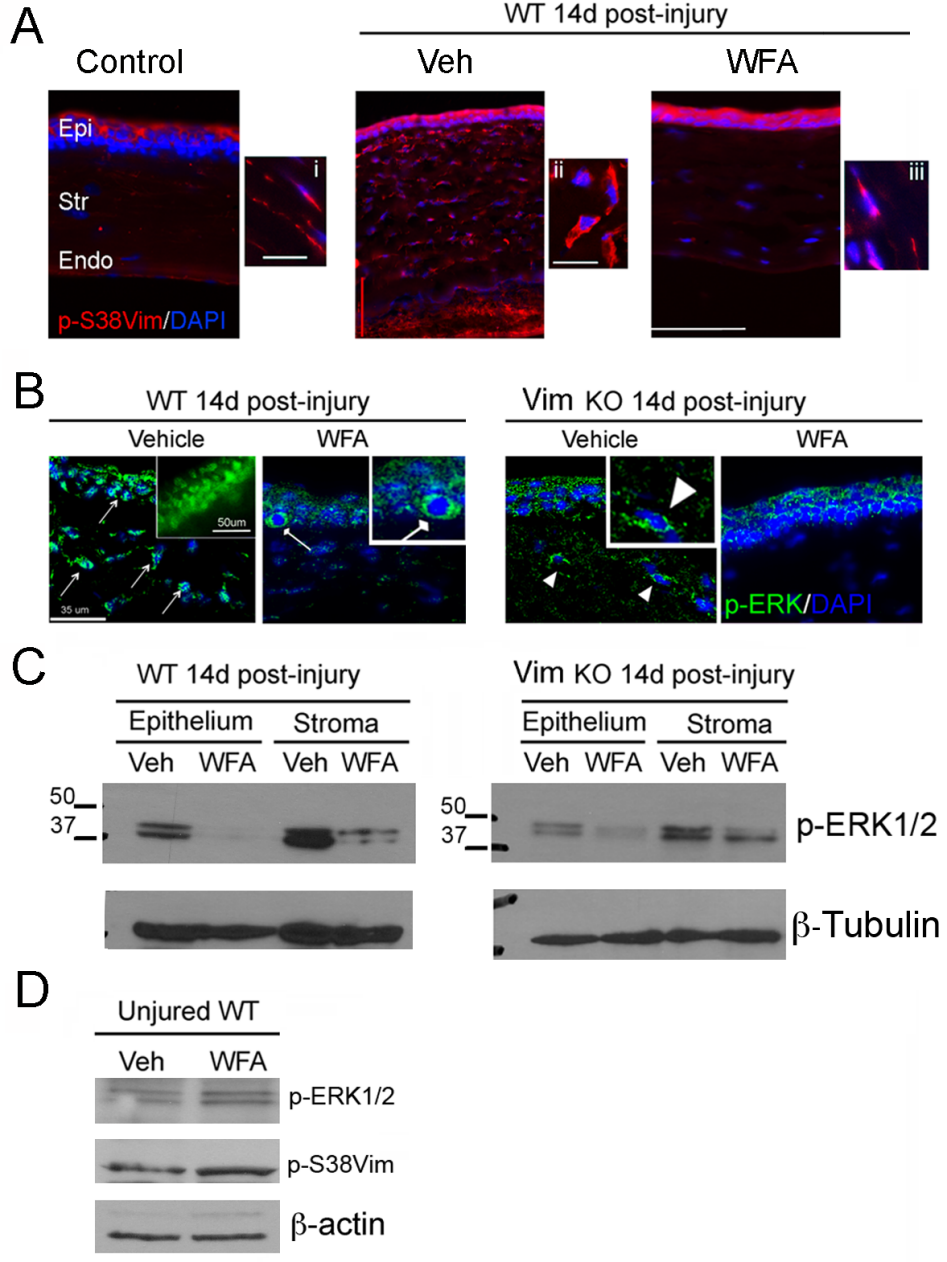
WFA blocks activation of the ERK1/2 pathway *in vivo* by targeting pSer38vim expression. Anesthetized mice were subjected to bilateral corneal alkali injury. Injured mice were treated with DMSO (Vehicle) or WFA (2 mg/kg solubilized in DMSO) on the day of injury and every subsequent day by intraperitoneal injection for 14 days. (A) Immunohistochemistry analysis of WT corneal sections stained with pSer38vim antibody (red) and DAPI (blue). Epi: Epithelium; Str: Stroma; End: Endothelium. Enlarged panels: 60X magnification. (B) Immunohistochemistry analysis of WT and Vim KO samples stained with anti-pERK1/2 antibody (Green) and DAPI (blue). Enlarged panel: 60X magnification of WT-Vehicle sample, showing nuclear expression pERK1/2 (Green). Pointed-ended arrows indicate nuclear localization of pERK1/2 in epithelial and stromal cells of WT-Vehicle samples. Square-ended arrow indicates cytoplasmic localization of pERK1/2 in the basal layer of the epithelium of WT-WFA samples. Arrowhead indicates partial cytoplasmic localization of pERK1/2 in stromal cells of Vim KO-Vehicle samples. (C) Western blot analysis of epithelial and stromal extracts from corneas of mice 14 d post-injury. Equal amount of total protein extracts were fractionated by SDS-PAGE and blots were probed with anti-pERK1/2 antibody. β-tubulin was used as loading control. (D) Western blot analysis of corneal extracts from uninjured WT mice treated for 7 d with WFA (2 mg/kg/d) or vehicle (DMSO). Total protein extracts were fractionated by SDS-PAGE and blots were probed with anti-vimentin, and anti-pERK1/2 antibodies. β-actin antibody was used as loading control.

To translate our cell culture findings towards an understanding of how WFA attenuates fibrosis *in vivo*, while at the same time WFA promotes regenerative healing after injury [34], we investigated expression of pSer38vim and pERK1/2 expression in the alkali injury model of corneal fibrosis. Groups of injured wild-type (WT) and vimentin-deficient (Vim KO) mice were injected intraperitoneally with vehicle or WFA (2 mg/kg/d) every day for 14 days and eyes were collected for analysis. We previously established that by 7 days post injury there was clear evidence of WFA-induced downregulation of fibrotic markers that by 14 days showed significant functional improvement of corneal clarity. Injured Vim KO mice also showed functional improvement of corneal clarity in this injury model [34]. When investigated by immunostaining, WT mice showed strong upregulation of pSer38vim expression in injured Veh-treated corneas that was found localized in the stroma to enlarged myofibroblastic cells (Fig 10A, panel ii, red) [34]. WFA treatment caused a downregulation of pSer38vim expression and restored a more spindle-like shape to stromal cells, which appeared similar in shape to uninjured controls (Fig 10A, compare panels i and iii). The pERK1/2 staining profile in WT injured mice showed prominent expression in the vehicle-treated corneas, with pERK1/2 showing predominant nuclear localization in both epithelial cells and stromal fibroblasts/myofibroblasts (Fig 10B, green, arrows). However, in WFA-treated corneas pERK1/2 expression was reduced and mainly found as perinuclear/cytoplasmic staining in basal cells of epithelium (Fig 10B, rounded-headed arrow). The stroma of corneas showed very little pERK1/2 staining. Interestingly, pERK1/2 expression in WT-WFA treated corneas was similar to that of injured Vim KO corneas, where reduced pERK1/2 expression was also mainly confined to the perinuclear/cytoplasmic region of stromal cells (Fig 10B, arrowheads). Vim KO mice treated with WFA showed potent downregulation of pERK1/2 expression, with stroma being the most prominently affected site. Western blot analysis corroborated the staining results and revealed that pERK1/2 expression was very high in the stroma of the WT-Veh corneas compared to Vim KO-Veh corneas, and WFA downregulated pERK1/2 expression in both WT corneal epithelium and stroma (Fig 10C). We also found that in injured Vim KO mice considerably reduced levels of pERK1/2 were found in vehicle-treated corneas in comparison to injured WT corneas, and with WFA treatment, there was altogether much less detectable pERK1/2 (Fig 10C). Finally, we wished to establish how uninjured mice responded to WFA treatment because the uninjured stroma expresses only naïve keratocytes having very little pSer38vim expression (Fig 10A). WT uninjured mice were injected with 2 mg/kg/d WFA for 7 d and western blot analysis of corneal tissues was performed. WFA treatment did not affect pERK1/2 expression levels compared to vehicle treatment. Moreover, the expression of pSer38vim also remained unchanged with drug treatment (Fig 10D). This showed that WFA was clearly targeting the activated states of injury when myofibroblast differentiation occurs and correlated with high pSer38vim expression. Taken together, our *in vivo* findings demonstrate firstly, that under a chronic treatment paradigm, the targeting of pSer38vim by WFA in myofibroblasts downregulates the differentiation of these cells *in vivo*. Secondly, WFA also attenuates ERK signaling in corneal fibroblasts/myofibroblasts, and this effect is corroborated by our genetic model showing that in Vim KO mice corneal injury also produces lower levels of pERK1/2 that remain mostly cytoplasmic.

## Discussion

Corneal fibroblasts and myofibroblasts express vimentin, and thus, it was not previously known whether vimentin overexpression *per se* governs their sensitivity to the anti-fibrotic drug WFA. Here using cell-spreading assays, we have discovered that myofibroblasts through acquisition of soluble pSer38vim makes them inefficient at incorporating this vimentin isoform into growing vimentin IFs, and consequentially, sensitizes myofibroblasts over fibroblasts to WFA inhibitory activity. Our results were also confirmed *in vivo*, showing fibrotic corneas of injured mice express high levels of pSer38vim, which is effectively targeted by WFA and downregulated to promote regenerative healing [34]. Taken together, our findings illuminate a novel mechanism of WFA that identifies pSer38vim as a key corneal biomarker and a target for anti-fibrosis.

Cell spreading involves extensive cytoskeletal remodeling of the actomyosin and intermediate filament networks, a process during which vimentin undergoes structural remodeling. We employed Blebb to perturb myosin cytoskeleton and indirectly cause vimentin filament depolymerization as a means to increase levels of sVim [44] for chemical conjugation with WFA [37], but found that this approach does not sensitize myofibroblasts to WFA. Yet, Blebb treatment when combined with overexpression of vimentin caused cells to become highly sensitive to WFA even though transfected vimentin extensively integrated into the endogenous cytoskeleton. Given that both myosin perturbation [48] and vimentin expression levels [10] can affect the spatial organization of FAs, we postulate that pre-existing endogenous filaments may release soluble subunits at FAs, that with transfected sVim may have reached a threshold that becomes prone to phosphorylation causing their heightened sensitivity to WFA. While this mechanism remains to be proven, our insights into the contextual targeting of sVim by WFA became more apparent when we examined a more physiological differentiation process undertaken by fibroblasts in becoming converted to myofibroblasts by cell passage.

Fibroblasts that had differentiated into myofibroblasts showed increased abundance of pSer38vim in the soluble pools, which suggested that a serine kinase may be co-activated during this differentiation process. Several kinases (Rho-kinase, PKC, PKA, AKT, CAMKII, Aurora-B and PAK) can phosphorylate vimentin at serine 38 in serum-treated fibroblasts [30], whereas serine 71 phosphorylation is predominantly mediated by Rho-kinase [49], and, in late mitotic cells, Rho-kinase causes vimentin reorganization specifically at the cleavage furrow [49]. Interestingly, serine 71 phosphorylation by ROCK promotes vimentin filament assembly (e.g. chemotactic migration to sphingolipid stimulation) [50], which is unique given the general consensus that phosphorylation at most known other sites causes vimentin filament disassembly [25] [26]. Although, early passage fibroblasts assemble vimentin efficiently during cell spreading possibly through ROCK-mediated serine 71 phosphorylation, its increased expression caused by WFA would effectively dampen the rate of spreading. However, in late passage cells the serine 38 phosphorylation of vimentin is the critical target of WFA and likely to be a breaking mechanism to modulate filament assembly. These data then implicate that fibroblasts and myofibroblasts that inherently express different levels of phosphorylated vimentin afford these respective cells with a dynamic pool of soluble precursors to respond to cellular cues in different manners. For instance, in 3T3 fibroblasts phosphorylation of vimentin at serine 38 promotes lamellipodia formation, and thus, motile cells inherently attenuate this phosphorylation event during initiation of cell polarization [30]. We are prompted to propose that a deficit in recruitment of sVim into growing vimentin IFs in myofibroblasts occurs because of increased levels of pSer38 soluble vimentin isoforms. Although we have not investigated kinetics of lamellipodia formation in myofibroblasts, the levels of soluble pSer38vim may also help explain the increased migratory rates of corneal fibroblasts over α-SMA-expressing myofibroblasts [51] [52].

Fibroblasts use FAs to link the cell membrane to the underlying matrix by recruiting intermediate sized vimentin IFs to developing FAs, where the vimentin-associated linker protein FlnA is essential to this process [43]. We discovered that FlnA although expressed equally in fibroblasts and myofibroblasts does not directly respond to WFA, ruling out its direct involvement in the mode of action of WFA. On the other hand, vimentin perturbation by WFA prevents the coalescence of vimentin IFs into developing FAs in mouse embryonic fibroblasts [6]. However, that prior study did not examine whether phosphorylation status of vimentin mediated this drug effect. Potentially, focal adhesion kinase (FAK) may be involved in vimentin phosphorylation as it acts as a mechanosensor linking adhesion-signaling to downstream targets such as ERK, and deficits in vimentin or plectin can downregulate FAK as well pERK expression causing impaired cell migration [11]. Moreover, fibroblast-specific FAK-deficiency in mice subjected to dermal injury showed reduced fibrosis and scarring due to mechanosensory deficits conveyed by an impaired ERK signaling mechanism [53]. Vim KO mice also show deficits in myofibroblast migration into dermal injury sites [13] and Vim KO mice have attenuated ERK activation in peripheral injured neurons [20], as well as in experimental retinal gliosis [54]. Collectively, these findings illuminate the importance of the vimentin-ERK axis in injury paradigms, where overexpression/overactivation of this complex is thought to drive fibrotic scar formation. This idea is corroborated by our findings showing injured Vim KO mouse corneas heal with significantly reduced fibrosis [34], and this corneal repair phenotype in vimentin deficiency reveals also downregulated pERK expression.

Although both WFA and Epox induce pSer38vim hyperphosphorylation in myofibroblasts and not in fibroblasts, their precise targeting mechanisms are quite different. WFA did not affect the levels of sVim or cause changes in polyubiquitinated proteins, whereas Epox induced reduction of sVim levels and stabilization of polyubiquitinated proteins by blockade of the 20S proteasome. Proteasome inhibition causes transcriptional repression of vimentin [55]; however, this latter mechanism does not explain the rapid loss of sVim with Epox treatment selectively in myofibroblasts, but not in fibroblasts. One could speculate that Epox may stabilize/activate a kinase that would induce pSer38vim hyperphosphorylation, but at the same time, the drug also causes sVim to become redistributed through polymerization into aggresomes [56], but this hyperphosphorylated pSer38vim would remain soluble because it is not integrated into vimentin IFs [30]. These mechanistic differences between WFA and Epox underscore the greater selectivity of WFA for targeting cell migration and in anti-cancer drug development than classical proteasome inhibitors [57].

Phosphorylated sVim is a chaperon for pERK1/2 that targets this kinase to sites of injury in peripheral neurons. We show for the first time that pSer38vim may also function as a chaperon for pERK1/2 in activated corneal fibroblasts and myofibroblasts. However, while no apparent differences in basal pERK1/2 levels between fibroblasts and myofibroblasts could be documented, instead, we found that WFA treatment preferentially increases pERK1/2 activation in myofibroblasts via novel cytoplasmic complexes that prevents pERK’s nuclear localization. It is possible that the hyperphosphorylated pSer38vim-bound pERK cytoplasmic complex distributes in other sub-cellular domains and has novel functions, which remain to be elucidated.

Attenuation of pERK expression mediated by vimentin in response to WFA underlies a novel anti-fibrotic mechanism that was previously not recognized. Apparently, injured Vim KO mice treated with WFA also show reduction of pERK expression, although this reduction is more visible in the corneal stroma. As such, this result would implicate another protein target of WFA, which is likely desmin. We previously showed that desmin is induced in injured Vim KO corneas and in WFA treated mice, desmin shows lowered expression and this protein shows retarded electrophoretic mobility by western blot analysis [34] that would suggest phosphorylation. It remains to be determined what form of desmin interacts with pERK and how this might mediate potential changes in pERK expression and/or localization. Finally, we show that in absence of an injury pSer38vim and pERK may exist in complexes that are insensitive to WFA, indicative of selectivity of WFA for targeting pSer38vim that becomes overexpressed during fibrosis. Taken together, we propose that overexpressed soluble pSer38vim is a novel biomarker of corneal fibrosis, which is a novel finding that will need validation in other models and in humans.

## Supporting Information

S1 Fig Western blot analyses of vimentin phosphorylation at serine residues 71, 72 and 82 in RbCF2 and RbCF10 cells. (TIF)

S2 Fig Western blot analyses of vimentin phosphorylation at serine residues 71, 72 and 82 in RbCF2 and RbCF10 cells in repeat experiments. (TIF)
