# Supplementary figures and images for: Vimentin Phosphorylation Underlies Myofibroblast Sensitivity to Withaferin A In Vitro and during Corneal Fibrosis
Source: PLoS One. 2015 Jul 17;10(7):e0133399. doi: 10.1371/journal.pone.0133399 (PMC4506086; doi:10.1371/journal.pone.0133399)

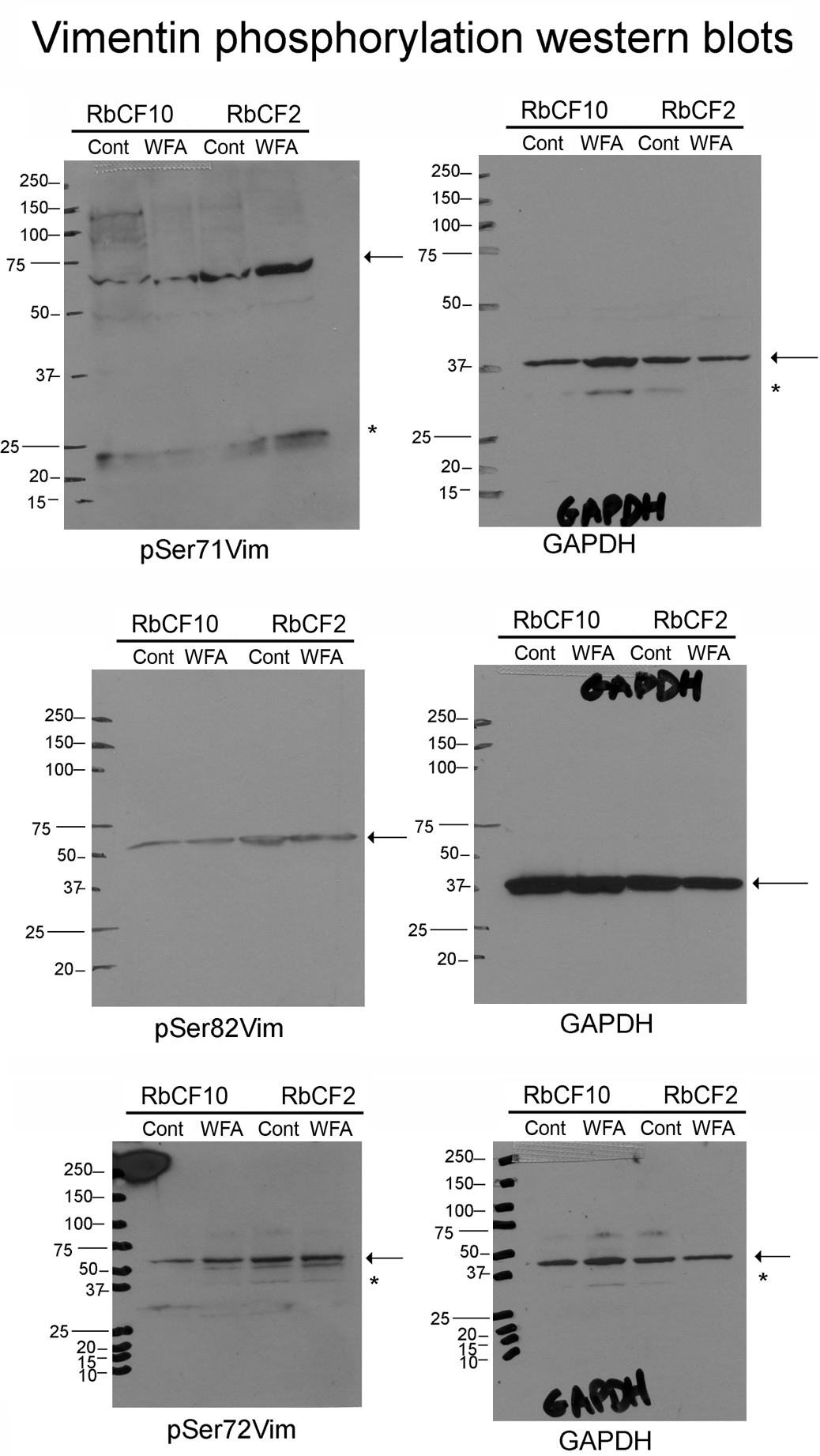

Supplement: S1 Fig — (TIF) [file pone.0133399.s001.tif]

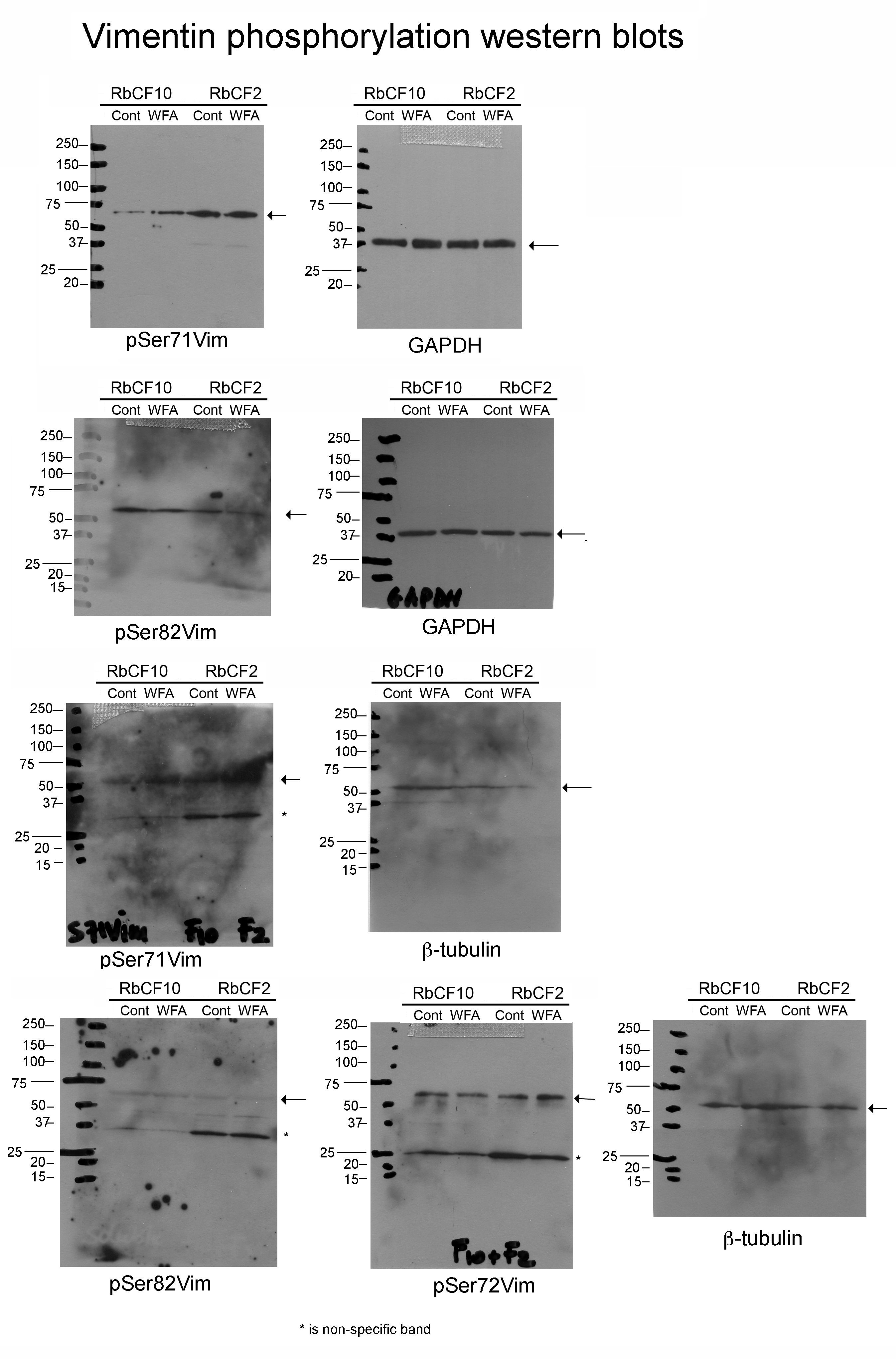

Supplement: S2 Fig — (TIF) [file pone.0133399.s002.tif]
